# Seasonality in Diffusive Methane Emissions Differs Between Bog Microforms

**DOI:** 10.1111/gcb.70372

**Published:** 2025-07-25

**Authors:** Katharina Jentzsch, Elisa Männistö, Maija E. Marushchak, Tabea Rettelbach, Lion Golde, Aino Korrensalo, Joshua Hashemi, Lona van Delden, Eeva‐Stiina Tuittila, Christian Knoblauch, Claire C. Treat

**Affiliations:** ^1^ Alfred Wegener Institute (AWI) Helmholtz Center for Polar and Marine Research Potsdam Germany; ^2^ Institute of Environmental Science and Geography University of Potsdam Potsdam Germany; ^3^ School of Forest Sciences University of Eastern Finland Joensuu Finland; ^4^ Department of Biological and Environmental Science University of Jyväskylä Jyväskylä Finland; ^5^ Department of Environmental and Biological Sciences University of Eastern Finland Kuopio Finland; ^6^ Institute of Geosciences University of Potsdam Potsdam Germany; ^7^ Fachbereich III Umweltingenieurwesen—Bau Berliner Hochschule für Technik Berlin Germany; ^8^ Natural Resources Institute Finland Joensuu Finland; ^9^ Department of Earth System Sciences University of Hamburg Hamburg Germany; ^10^ Center for Earth System Research and Sustainability University of Hamburg Hamburg Germany; ^11^ Department of Agroecology Aarhus University Aarhus Denmark

**Keywords:** boreal, chamber measurements, methane, microtopography, peatland, subarctic, upscaling, vegetation removal experiment

## Abstract

Wetlands are the largest natural source of atmospheric methane (CH_4_), but substantial uncertainties remain in the global CH_4_ budget, partly due to a mismatch in spatial scale between detailed in situ flux measurements and coarse‐resolution land surface models. In this study, we evaluated the importance of capturing small‐scale spatial heterogeneity within a patterned bog to better explain seasonal variation in ecosystem‐scale CH_4_ emissions. We conducted chamber‐based flux measurements and pore water sampling on vegetation removal plots across different microtopographic features (microforms) of Siikaneva bog, southern Finland, during seasonal field campaigns in 2022. Seasonal and spatial patterns in CH_4_ fluxes were analyzed in relation to key environmental and ecological drivers. High‐resolution (6 cm ground sampling distance) drone‐based land cover mapping enabled the extrapolation of microscale (< 0.1 m^2^) fluxes to the ecosystem scale (0.75 km^2^). Methane emissions from wetter microforms (mud bottoms and hollows) closely followed seasonal changes in peat temperature and green leaf area of aerenchymatous plants, while emissions from drier microforms (high lawns and hummocks) remained seasonally stable. This constancy was attributed to persistently low water tables, which moderated environmental fluctuations and reduced seasonality of CH_4_ production, CH_4_ oxidation and plant‐mediated transport. The strong spatial pattern in CH_4_ emissions and their seasonal dynamics made both the magnitude and seasonal cycle of ecosystem‐scale emissions highly sensitive to the areal distribution of microforms. Our findings underscore the need to integrate microscale spatial variability into CH_4_ modelling frameworks, as future shifts in peatland hydrology due to climate change may alter the balance between wet and dry microforms—and with it, the seasonal and annual CH_4_ budget.

## Graphical abstract

This study shows that small‐scale landscape features within a boreal bog strongly influence seasonal patterns of methane emissions. By combining ground‐based measurements with high‐resolution drone mapping, the research highlights that wetter areas dominate methane emissions in spring and summer, while emissions from drier areas gain importance in the fall. These contrasting seasonal dynamics highlight the need to incorporate fine‐scale spatial variation into climate models predicting methane release from wetlands.

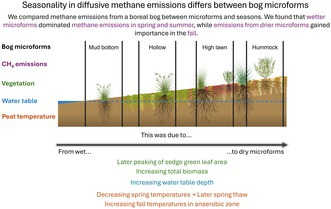

## 1 Introduction

Wetlands are the largest natural source of methane (CH_4_) to the atmosphere, accounting for approximately 30% of global emissions (Saunois et al. 2020). These emissions are expected to undergo substantial changes due to climate‐driven shifts in temperature and hydrology, with important implications for the global CH_4_ budget. Such changes are projected to be most pronounced at high latitudes, where peatlands are the dominant wetland type (Rantanen et al. 2022). Process‐based numerical models are commonly used to predict future CH_4_ emissions from peatlands, but their accuracy is limited by an incomplete understanding of the underlying biogeochemical processes and their environmental and ecological controls (Saunois et al. 2016, 2020).

One major limitation of current process‐based models is their inadequate representation of the seasonal dynamics of peatland CH_4_ emissions. In particular, cold‐season emissions are often underestimated (Ito et al. 2023; Treat et al. 2018). Methane emissions from northern peatlands typically peak during the summer months, when solar radiation, air and peat temperatures and primary productivity are at their highest (Dise 1993; Jackowicz‐Korczyński et al. 2010; Long et al. 2010; Moore and Knowles 1990; Saarnio et al. 1997). During this period, increased microbial activity and a high availability of labile carbon substrates promote CH_4_ production, while efficient plant‐mediated transport facilitates its release to the atmosphere. In contrast, the drivers of CH_4_ emissions during winter and transitional shoulder seasons remain less well understood. Although emissions are generally lower during these periods, they are often higher than expected based on the prevailing low temperatures and reduced plant activity (Ito et al. 2023; Treat et al. 2018).

Another key limitation of large‐scale CH_4_ models is their coarse spatial resolution (e.g., 0.5° grids), which typically allows only a binary distinction between wetland and upland areas (Albuhaisi et al. 2023). In contrast, the most pronounced differences in CH_4_ fluxes occur at the sub‐metre microscale, where variations in environmental variables such as water table depth and soil temperature are highest (Waddington and Roulet 1996). Ombrotrophic bogs, peatland ecosystems that rely solely on precipitation and atmospheric deposition for water and nutrient inputs, exhibit particularly high microscale heterogeneity due to their pronounced microtopography. This includes a mosaic of surface types (microforms) ranging from open pools and wet hollows to intermediate lawns and drier hummocks (Pakarinen 1995; Seppä 2002). Once established by water flowing along the slope of the raised bog, this topographic variation is maintained through feedbacks between moisture conditions, plant community composition, decomposition rates and peat accumulation (Couwenberg and Joosten 2005; Seppä 2002).

This microtopographic variability drives substantial spatial differences in CH_4_ cycling by influencing all three components of CH_4_ fluxes: production, oxidation and transport. W affects CH_4_ production and oxidation by regulating the thickness of the aerobic acrotelm layer (Dise et al. 1993; Ström and Christensen 2007). Vascular plants also play a central role, either enhancing or suppressing CH_4_ emissions. They can increase emissions by supplying labile carbon through root exudates and plant litter and by facilitating CH_4_ release through plant‐mediated transport. Conversely, aerenchymatous plants also allow for oxygen leakage into the rhizosphere, thereby promoting CH_4_ oxidation (Joabsson et al. 1999). The net effect varies by species depending on their root exudation profiles (Dorodnikov et al. 2011; Ström et al. 2003), transport efficiency (Korrensalo et al. 2022; Schimel 1995) and capacity for rhizospheric oxidation (Ström et al. 2005). As a result, CH_4_ emissions are typically highest from wet microforms such as bare peat surfaces, hollows and lawns, while drier hummocks often show reduced emissions or may even act as net CH_4_ sinks (Bubier et al. 1993, 1995; Frenzel and Karofeld 2000; Heikkinen et al. 2002; Laine et al. 2007; Moore and Knowles 1990; Waddington and Roulet 1996).

Climate change is increasing air temperatures and altering precipitation patterns, especially in northern high‐latitude regions, with profound implications for greenhouse gas dynamics in peatlands, particularly CH_4_ emissions (Hopple et al. 2020). Although precipitation is projected to increase in boreal regions due to climate change, elevated evapotranspiration is expected to result in overall drier soil conditions (Fekete et al. 2010; IPCC 2023). These hydrological changes may lower water tables in boreal bogs, leading to shifts in vegetation composition towards communities dominated by dwarf shrubs and increases in plant productivity (Breeuwer et al. 2009; Bubier et al. 2003; Holmgren et al. 2015; Laine et al. 1995; Kokkonen et al. 2019) with potential implications for microform distribution. Climate change is likely to affect both the total magnitude of CH_4_ emissions from boreal peatlands and their seasonal dynamics. Rising air temperatures are projected to cause earlier spring thaw and snowmelt, along with delayed soil freezing in the fall, thereby lengthening the growing season and potentially enhancing plant productivity and CH_4_ emissions (Euskirchen et al. 2006; Helbig et al. 2017). However, increased frequency and severity of droughts may offset these productivity gains (Lund et al. 2012) and result in a temporary net carbon loss through increased aerobic decomposition (Fenner and Freeman 2011; Rinne et al. 2020). Warming is projected to be most pronounced in winter, potentially reducing snow cover as more precipitation falls as rain (Kellomäki et al. 2010; Mudryk et al. 2014). A thinner snowpack may lessen insulation, leading to deeper soil freezing (Brown and DeGaetano 2011; Campbell et al. 2010; Zhang 2005). However, snow cover trends remain uncertain, with some regions showing increased winter accumulation (Cohen et al. 2012; Mudryk et al. 2014). More frequent freeze–thaw cycles may also trigger episodic CH_4_ release from beneath frozen soil layers (Liu et al. 2024; Yang et al. 2022).

The effects of climate change on CH_4_ emissions will also depend on microscale spatial variability. Although warmer peat temperatures may enhance CH_4_ production potential, emissions from drier microforms such as hummocks are expected to decline due to thickening of the aerobic surface layer, which promotes CH_4_ oxidation (Strack et al. 2008). In contrast, wetter microforms such as hollows may maintain high CH_4_ emissions. In these areas, subsidence of less rigid peat could help preserve high water tables despite reductions in overall water storage (Whittington and Price 2006).

Despite growing recognition of the seasonal and spatial variability in CH_4_ emissions from boreal peatlands, the interaction between these two dimensions remains poorly understood. A thorough understanding of current seasonal dynamics is essential for predicting how CH_4_ emissions will respond to changes in the length and timing of the growing season, snow cover dynamics and soil freeze–thaw patterns under future climate scenarios. At the same time, predicted changes in hydrology and vegetation are unlikely to affect peatland surfaces uniformly. Instead, their impacts are expected to vary across the distinct microforms that characterize ombrotrophic bogs. Understanding the relative contributions of these microforms to the overall peatland CH_4_ flux throughout the year is therefore critical. These insights are essential for improving the accuracy of ecosystem‐scale CH_4_ budgets and reducing uncertainties in future CH_4_ emission projections from boreal peatlands.

The aim of this study was to assess how microscale spatial heterogeneity within a patterned boreal bog influences seasonal variability in ecosystem‐scale CH_4_ emissions. Specifically, we investigated how microforms contribute to differences in CH_4_ fluxes across seasons and how these patterns scale up to the ecosystem level. To achieve this, we pursued the following objectives:

1. *Quantify spatial and seasonal variation* in key environmental variables and CH_4_ fluxes across the microtopographic gradient (mud bottoms, hollows, high lawns and hummocks) of Siikaneva bog in southern Finland.
2. *Identify the environmental and ecological controls* on spatial and seasonal CH_4_ flux variability across microforms.
3. *Upscale CH*_*4*_ *fluxes* from microforms to the ecosystem level to evaluate the importance of spatial heterogeneity for interpreting ecosystem‐scale CH_4_ dynamics.

## 2 Materials and Methods

Measurements for this study were conducted at Siikaneva bog, Southern Finland, in four seasonal field campaigns in spring (May), summer (July) and fall (September and October) 2022 (Figure S1). Chamber measurements of CH_4_ fluxes were taken at microtopographical‐scale vegetation removal plots along with pore water samples for concentrations of dissolved CH_4_ and dissolved organic carbon (DOC). These vegetation removal experiments were designed to quantify the seasonal effects of *Sphagnum* mosses and vascular plants on CH_4_ fluxes. By combining flux measurements with pore water chemistry and environmental variables—including leaf area index (LAI), peat temperatures and water table depth (WTD)—we were able to evaluate vegetation effects on CH_4_ production, oxidation and transport processes. Finally, the chamber fluxes were upscaled to the bog level using a microtopographical classification derived from drone imagery. The data set is publicly available at https://doi.org/10.1594/PANGAEA.971358 (Jentzsch et al. 2024b).

### 2.1 Study Site

**FIGURE 1:**
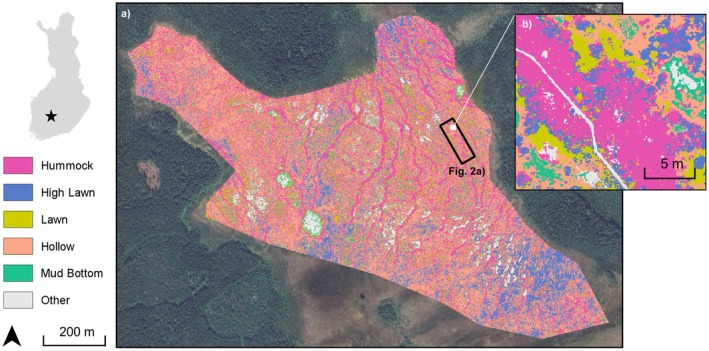
Location and landcover classification of Siikaneva bog, focussing on the microforms addressed in this study. Open water, islands of mineral soil and boardwalks are grouped under the category ‘Other’. The classified area in panel (a) represents the full extent of Siikaneva bog. Chamber measurement plots for CH_4_ fluxes were located in the black‐outlined rectangle and are shown in detail in Figure 2. Panel (b) presents an enlarged view of the landcover classification in a 20 × 20 m subarea within this chamber measurement area. Map lines delineate study areas and do not necessarily depict accepted national boundaries.

Siikaneva bog is the ombrotrophic part of the Siikaneva peatland complex, located in Southern Finland at 61°50′ N and 24°12′ E and 160 m a.s.l. (Figure 1). The annual precipitation in the area is 688 mm, of which about one third falls as snow (Riutta et al. 2020), the average annual temperature is 4.1°C, and average temperatures in January and July are −6.5°C and 16.4°C, respectively (30‐year average [1993–2022] from the nearby Juupajoki‐Hyytiälä weather station).

**FIGURE 2:**
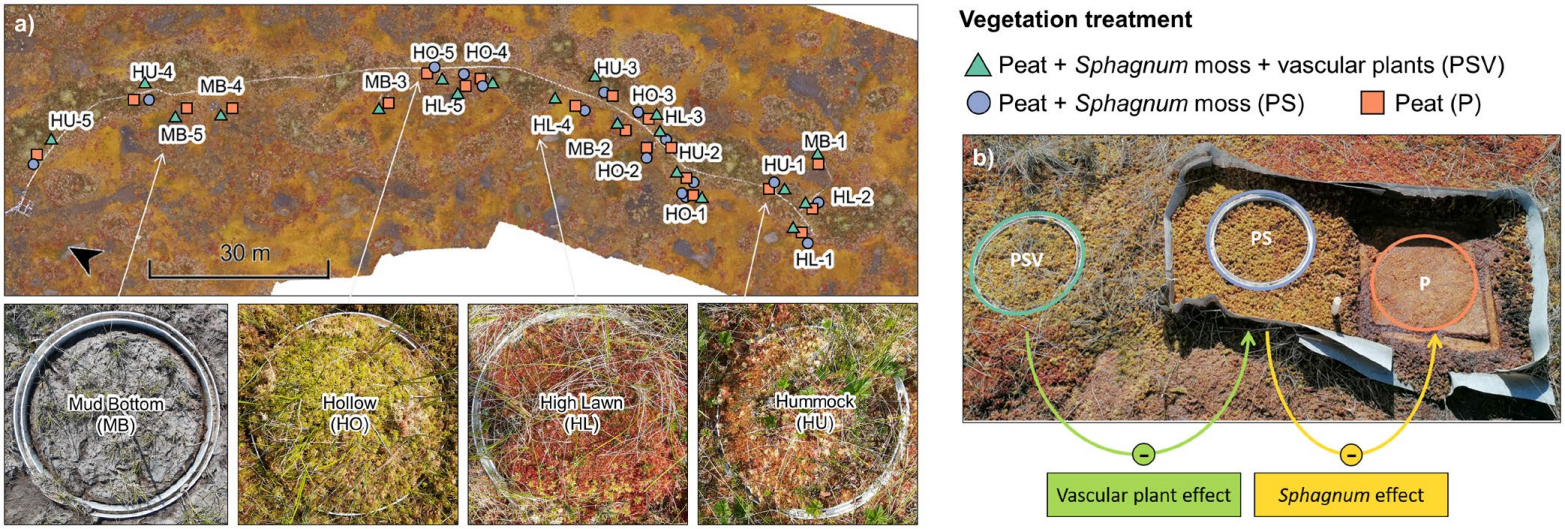
Examples of plots for chamber measurements with intact vegetation (PSV) representing the four studied microforms. The map shows the location of the five spatial replicates (numbers 1–5) of a vegetation removal experiment per microform (a). Close in on a vegetation removal experiment in a hollow indicating the calculations used to derive the effects of vascular plants and of the *Sphagnum* moss layer on CH_4_ fluxes (b, figure 1c from Jentzsch et al. 2024a).

Siikaneva bog has a pronounced microtopography ranging from open‐water pools and low‐lying bare peat surfaces to wet hollows and intermediate lawns to drier and higher hummocks. For the plot‐scale measurements, we classified four microforms: mud bottoms, hollows, high lawns and hummocks, based on their surface height and associated WTD and their characteristic plant communities (Korrensalo, Männistö, et al. 2018). In total, these microforms cover 81% of the bog area (Figures 1 and 2, Table 2). In the wettest microform, the mud bottoms, the moss layer is missing, and *Rhynchospora alba* is often the only plant growing. In the hollows, the moss layer consists of *Sphagnum cuspidatum* and *Sphagnum majus* , and the vascular plant cover is dominated by aerenchymatous sedges, such as *Carex limosa* , *R. alba* and *Scheuchzeria palustris* . On high lawns, *Sphagnum magellanicum* and *Sphagnum rubellum* make up most of the moss layer, and *Eriophorum vaginatum* is the dominant vascular plant species. On hummocks, *E. vaginatum* also occurs, but dwarf shrubs, such as *Andromeda polifolia* , *Calluna vulgaris* and *Empetrum nigrum*, prevail together with *Sphagnum fuscum* and *S. rubellum* (Table S1).

### 2.2 Microtopographical‐Scale Measurements

A detailed description of the measurement and sampling design on the microtopographical scale as well as of the analytical methods, data processing and quality control steps is given in Jentzsch et al. (2024a). Per microform, our study design comprised five spatial replicates of a vegetation removal experiment (Figure 2a). Each of the spatial replicates consisted of a plot cluster including one control plot with intact vegetation (Peat + *Sphagnum* moss + vascular plants [PSV]), one plot with the vascular plants removed and only the moss layer remaining (PS), and one bare peat plot with all vegetation removed [P] (Figure 2b). There were no PS plots in the mud bottoms, as the moss layer is naturally missing from this microform. This resulted in a total of 20 plot clusters across the microforms, containing 55 individual plots for chamber measurements. When the vegetation removal experiment was established in 2016, to create the PS and P treatments, all vascular plants were clipped from an area of 0.5 m^2^, and the area was surrounded by polypropylene root barrier fabric 70 cm deep into the ground to keep roots from growing back into the area from the sides. Ever since, any newly growing vascular plants have been gently pulled out with their roots. To create the P treatment, within the vascular plant removal area, about 40 × 40 cm of the 4–5 cm thick living *Sphagnum* moss layer was cut out and placed on net fabric in a frame that could be lifted aside, exposing the bare peat.

CH_4_ fluxes between peat and the atmosphere were estimated by placing a transparent chamber connected to an in‐line gas analyser (Licor LI‐7810 or LGR Microportable Greenhouse Gas Analyzer [MGGA]) on circular collars enclosing an area of 0.074 m^2^ at each of the plots. Collars for chamber measurements were permanently installed at the PSV and PS plots, while at the P plots the pre‐cut moss layer was lifted aside and a collar was placed underneath only shortly before each chamber measurement. For each chamber closure, we calculated the mean diffusive CH_4_ flux as the slope of a linear fit to the CH_4_ concentration timeseries recorded in the chamber headspace after excluding potential periods of initial disturbance caused by chamber placement and episodic ebullition events from the timeseries. In this study, we consider diffusive CH_4_ fluxes only because the observed ebullition events were likely largely triggered by the chamber placement and might therefore not be representative of the ebullitive flux under undisturbed conditions. The prevalence of ebullition events by measurement campaign, microform and vegetation treatment, however, gave us some indication of the environmental conditions under which CH_4_ ebullition is most likely to occur (Figure S2). The effects of vascular plants on CH_4_ fluxes were calculated as the difference between the fluxes measured at the control plots (PSV) and at the plots where vascular plants had been removed (PS treatment for hummocks, high lawns and hollows and P treatment for mud bottoms). The effect of the moss layer at the hummocks, high lawns and hollows was calculated as the difference between plots with moss (PS treatment) and without moss (P treatment).

During each field campaign, we sampled the pore water at 20 cm depth at the control plot and at the moss plot of each plot cluster. A subsample was filtered, acidified and analysed for DOC on a Shimadzu TOC‐L analyzer. The remaining sample was mixed with an equal volume of nitrogen gas to extract the CH_4_ dissolved in the pore water. The gas phase was then analysed for its CH_4_ concentration using Cavity Ring‐Down Spectroscopy (CRDS; Picarro G2201‐I Isotopic Analyzer with autosampler SAM). Corrections were applied for dilution during gas extraction and sample analysis. We report CH_4_ concentrations only when pore water was available for sampling; measurements were not considered when only gas could be extracted from the pore space.

Along with pore water sampling, we measured peat temperatures at 20 cm depth. Furthermore, we measured the WTD relative to the moss surface at each plot cluster, with negative values indicating water levels below the moss surface. To obtain the LAI for each vascular plant species inside the control plots, we estimated the green area of all species five times during the growing seasons by combining leaf counting and leaf area measurements with a LI‐3000 portable area meter and fitted log‐normal models to describe the seasonal dynamics of each species (Wilson et al. 2007). The total LAI of green vascular plants (LAItot), of green aerenchymatous plants (LAIaer), and of shrubs (LAIshrub) was calculated as the sum of the LAI of all vascular plants, all aerenchymatous species and all shrub species present in the measurement plot, respectively. Depending on the microform, the species contributing to LAIshrub were *A. polifolia* , *Betula nana* , *C. vulgaris* , *E. nigrum* , *Rhododendron tomentosum* , *Pinus sylvestris* , *Rubus chamaemorus* and *Vaccinium oxycoccos* . The aerenchymatous plants assessed for LAIaer included *C. limosa* , *Carex pauciflora* , *E. vaginatum* , *R. alba* , *S. palustris* and *Trichophorum cespitosum* .

### 2.3 Statistical Analyses

All statistical analyses for this study were done in the R environment (version 4.3.0; R Core Team 2021). We used linear mixed‐effects models to test whether the environmental variables as well as the effects of vascular plants and of the moss layer on the CH_4_ fluxes differed significantly between seasons and microforms. To explain the variation in the CH_4_ fluxes themselves as well as in the pore water data, we considered the vegetation treatment as an additional fixed effect in the models.

To build the models, we used the lmer function of the lme4 package (Bates et al. 2015) with restricted maximum likelihood and with a unique identifier for each measurement plot as a random effect, expressed as the combination of microform, spatial replicate and vegetation treatment. To assess the explanatory power of the fixed predictors and their interactions based on *F*‐tests and *p* values, we applied a type III ANOVA with the Kenward–Roger approximation to adjust the degrees of freedom using the anova function from the stats package (R Core Team 2021). We consider this approach as best suited for our slightly unbalanced data set that has one cell missing, which is the PS treatment for the mud bottom microform. We used the dredge function from the MuMIn (Bartoń 2010) package to identify the best combination of fixed predictors based on AICc, fit for relatively small sample sizes.

To identify significant differences (*p* < 0.05) between relevant individual combinations of season, microform and vegetation treatment, we applied the post hoc Tukey's HSD (honestly significant difference) test using the emmeans function of the emmeans package (Lenth 2017) to obtain the estimated marginal means and computed all simple main‐effect comparisons using the ‘simple’ argument in the pairs function.

We applied multiple linear regression also to identify the environmental variables controlling the CH_4_ fluxes from the different vegetation treatments as well as the vegetation effects on CH_4_ fluxes, again using the plot identifier as a random effect in the linear mixed‐effects models. As potential environmental controls (fixed effects), we considered the peat temperature at 20 cm depth, WTD, LAItot and LAIaer. All potential fixed predictors were standardized to account for their different units and their different scales, which in part differed by several orders of magnitude. To avoid multicollinearity, interactions between environmental variables were selected so that the variance inflation factor (VIF) of all potential fixed predictors remained below a value of 5. Again, we used the dredge function to identify the combination of fixed predictors that best described our observations. As it was previously shown that the explanatory power of models was increased when the different microforms were considered separately (Kettunen et al. 2000; Laine et al. 2007), we additionally built models for the individual microforms to highlight processes that might be obscured in the overall model.

To achieve normality of the residuals, the CH_4_ fluxes as well as the derived effect of vascular plants on CH_4_ fluxes had to be logarithmically transformed prior to statistical analysis. As negative values occurred both in the CH_4_ fluxes measured from hummocks as well as in the vascular plant effects, we used the pseudo‐logarithm to transform this data:

(1) $$\text{pseudo}\;\log_{10}\left(x\right)=\frac{\text{arsinh}\left(\frac{x}{2}\right)}{\ln\left(10\right)}=\frac{\ln\left(\frac{x}{2}+\sqrt{{\left(\frac{x}{2}\right)}^{2}+1}\right)}{\ln\left(10\right)}$$

Mean values μ reported in the text for the pseudo‐log‐transformed data were calculated based on the pseudo‐log‐transformed mean values $\bar{\mu }$ and transformed back to original scale using

(2) $$\begin{array}{c} \bar{\mu }=\text{pseudo}\;\log_{10}\left(\mu \right)\\ ↔\mu =10^{-\bar{\mu }}\times \left(10^{2\bar{\mu }}-1\right) \end{array}$$

The standard deviation sd reported in the text for the pseudo‐log‐transformed data was estimated based on the delta method using the backtransformed mean and the standard deviation of the pseudo‐log‐transformed data $\bar{\mathrm{sd}}$

(3) $$\begin{array}{c} \mathrm{sd}\left(f\left(x\right)\right)≅f^{\prime }\left(\mu \right)\mathrm{sd}\left(x\right)\\ ↔\mathrm{sd}\left(x\right)≅\mathrm{sd}\left(f\left(x\right)\right)/f^{\prime }\left(\mu \right) \end{array}$$

For

(4) $$\begin{array}{c} f\left(x\right)=\text{pseudo}\;\log_{10}\left(x\right)\\ f^{\prime }\left(x\right)=\frac{1}{\sqrt{x^{2}+4}\ln\left(10\right)} \end{array}$$

and therefore

(5) $$\begin{array}{c} \mathrm{sd}\left(x\right)≅\mathrm{sd}\left(\text{pseudo}\;\log_{10}\left(x\right)\right)\sqrt{\mu^{2}+4}\;\ln\left(10\right)\\ \text{or}\\ \mathrm{sd}≅\bar{\mathrm{sd}}\sqrt{\mu^{2}+4}\;\ln\left(10\right) \end{array}$$

We performed a principal component analysis (PCA) with all variables used in this study to visualize seasonal and spatial differences and similarities between our measurement plots using the function princomp from the stats package (R Core Team 2021).

### 2.4 Seasonal Upscaling of Chamber Measurements to the Ecosystem Scale

#### 2.4.1 Landcover Classification Using Drone Imagery and Calculations

To obtain high‐resolution remote sensing images of the study area for spatially upscaling our analysis, we conducted imaging surveys with an uncrewed aerial vehicle (UAV) in September 2022. We used a DJI Phantom 4 Multispectral UAV with six sensors: four 32 nm‐wide bands centred in the blue (median wavelength: 450 nm), green (560 nm), red (650 nm), and red‐edge (730 nm) ranges, as well as a 52 nm‐wide band centred in the near‐infrared (840 nm) range. Images from these five main sensors are stored as georeferenced 16‐bit GeoTIFFs. A sixth camera captured true‐colour JPEGs through a combined RGB sensor (red, green, blue). The JPEG images do not contain georeferences, however. We flew surveys at a constant altitude of 100 m a.g.l.

To process a multispectral orthomosaic of the collected data, we combined the images of the blue, green, red, red‐edge and near‐infrared sensors and used the photogrammetry software Pix4DMapper to compute the combined dataset. The resulting five‐band orthomosaic covers Siikaneva bog (approximately 0.75 km^2^) at 6 cm ground sampling distance (GSD) and spatial resolution (Figure 1).

For each band, we further calculated a standard‐deviation map and a ratio map to gather information on a pixel's surrounding area (spatially and spectrally) and to account for minor differences in sun irradiance throughout the survey area. For each pixel, we took into consideration its 9‐by‐9‐pixel neighbourhood and logged the standard deviation for each centre pixel into the standard‐deviation map for this band. For the ratio map, we divided each pixel value by the sum of all bands' pixel values and logged this to the new ratio map.

We used a Random Forest supervised classifier to segment the area of interest into eight different land cover classes: open water, mud bottom, hollow, lawn, high lawn, hummock, mineral soil and boardwalk. The training dataset consisted of 722 manually labelled points distributed over these eight classes. The input data to the Random Forest classifier consisted of the five multispectral bands of the computed orthomosaic, plus each band's standard‐deviation and ratio map (15 bands total). Our Random Forest was built with 95 decision trees (more trees did not significantly increase the mean classification accuracy). The classification's mean accuracy lies at 75.7% (Figure S3). The resulting land cover map can be seen in Figure 1. Table 2 gives an overview of the total area covered by each of the eight analysed land cover classes, including their uncertainties, calculated from the rate of misclassifications (the difference between 100% and the true‐positive value on the diagonal axis of Figure S3).

#### 2.4.2 Ecosystem‐Scale Emissions by Season

We used the land cover classification derived from the drone imagery to upscale the chamber CH_4_ fluxes from the microtopographical scale to the ecosystem scale. For this, we weighted the back‐transformed mean flux per microform by the relative contribution of the respective microform to the area of Siikaneva bog and derived the mean seasonal CH_4_ flux across the bog ecosystem. For consistency with earlier upscaling studies at Siikaneva bog (Alekseychik et al. 2021; Korrensalo, Männistö, et al. 2018) that considered lawns and high hummocks as additional microforms, we accounted for these microforms by assuming equal emissions from hummocks and high hummocks and by estimating the lawn emissions as the mean of hollow and high lawn emissions. We made this decision based on the values of LAIaer, which were shown to significantly affect CH_4_ fluxes and which were similar between hummocks and high hummocks and in between the hollow and high lawn values at the lawns (Korrensalo, Männistö, et al. 2018). Furthermore, the vegetation species present at the hummocks and the species present at the hollows and high lawns in our study were also found at the high hummocks and lawns, respectively (Table S1, table 1 in Korpela et al. 2020).

## 3 Results

### 3.1 Variation in Ancillary Data

#### 3.1.1 Variation in Environmental Variables

The seasonal variation in all considered environmental variables at the control plots with intact vegetation (PSV) differed significantly between microforms, as shown by interacting effects of measurement campaign and microform on the environmental variables; that is, for WTD (*F*_(9,165.69)_ = 21.888, *p* < 0.0001), peat temperature at 20 cm depth (*F*_(9,165.68)_ = 14.241, *p* < 0.0001), LAIaer (*F*_(9,167.91)_ = 24.498, *p* < 0.0001), LAIshrub (*F*_(9,151.62)_ = 7.539, *p* < 0.0001) and LAItot (*F*_(9,152.05)_ = 6.839, *p* < 0.0001).

**FIGURE 3:**
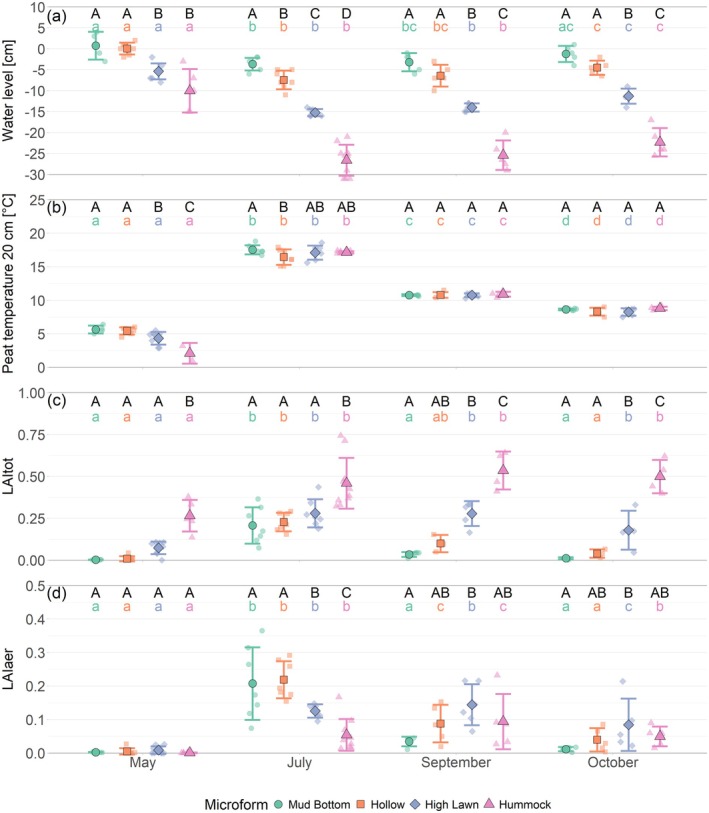
Mean and standard deviation of water level relative to the moss surface (a), peat temperature at 20 cm depth (b), total leaf area index (LAItot) (c) and leaf area index of aerenchymatous plants (LAIaer) (d) by microform and measurement campaign. Different capital letters indicate significant differences (*p* < 0.05) between microforms within one measurement campaign, and different small, coloured letters indicate significant differences between measurement campaigns for one microform.

The WTD showed a similar seasonal trend at all microforms, with the highest water levels occurring in May (−3.7 ± 5.0 cm), which dropped significantly until July (−14.5 ± 9.5 cm) and rose to significantly higher levels again until October (−9.8 ± 8.5 cm) (Figure 3a). In all seasons, the mean WTD deepened with increasing surface elevation, being shallowest at the mud bottoms, followed by the hollows, high lawns and deepest at the hummocks. In July, this water table gradient was statistically significant between all microforms (mud bottoms: −4.4 ± 2.1 cm, hollows: −8.2 ± 2.1 cm, high lawns: −15.6 ± 2.3 cm, hummocks: −27.3 ± 4.0 cm). Water table positions remained below the moss surface year‐round at the high lawns and hummocks, while the mean water table position was above the surface at the mud bottoms and hollows in May and at individual mud bottom plots in October. When the 4–5 cm of living moss layer was removed to build the P treatment, the water table was close to the bare peat surface also at the high lawns in May and at the hollows in September and October.

Peat temperatures at 20 cm depth across all microforms increased significantly between May (4.8°C ± 1.3°C) and July (17.1°C ± 0.8°C) and decreased significantly until September (10.8°C ± 0.3°C) and again until October (8.5°C ± 0.5°C) but remained significantly higher than the May temperatures (Figure 3b). The peat temperatures were uniform across all microforms during both fall campaigns, while in July, peat temperatures were significantly lower at hollows than at mud bottoms and in May, peat temperatures were highest at mud bottoms and hollows (5.6°C ± 0.5°C), intermediate at high lawns (4.5°C ± 0.8°C) and lowest at hummocks (2.1°C ± 1.3°C). Peat temperatures were significantly lower at the plots where all vascular plants had been removed to build the PS treatment (*t* (61) = 2.825; *p* = 0.0064). As the moss layer was removed from the bare peat treatments only for the duration of the chamber measurements, we assumed similar peat temperatures between both vegetation removal treatments.

Both LAIaer and LAIshrub, and thus LAItot, showed an overall seasonal trend of low LAI values in May, high values in July and September and intermediate values in October (Figure 3c,d). The timing of peak LAI, particularly of aerenchymatous plants, however, differed between the microforms due to their different species compositions (Table S1), with the highest LAIaer occurring later in the year at microforms with a lower water level. Maximum LAIaer was reached earliest at the mud bottoms and occurred 7, 32 and 36 days later on average at the hollows, high lawns and hummocks, respectively (Figure S4). LAIaer therefore dropped significantly between July and September at the mud bottoms and hollows, while at the high lawns, the significant decrease in LAIaer occurred only between September and October, and LAIaer was similar to July levels in October still at the hummocks. The spatial pattern in LAIaer therefore changed between seasons. In spring, values were similar and close to zero across all microforms. In summer, LAIaer was highest at bottoms and hollows, intermediate at high lawns and lowest at hummocks. In fall, LAIaer was significantly higher at the high lawns compared to the mud bottoms.

Despite the seasonally changing spatial pattern in LAIaer, a higher LAItot at drier compared to wetter microforms was maintained throughout the year. This was due to higher LAIshrub at deeper WTD of the microforms. LAIshrub remained close to zero year‐round at the mud bottoms and hollows but was significantly higher at the hummocks: the plant community of mud bottoms and hollows was strongly dominated by aerenchymatous plants (98% ± 2% and 88% ± 22% of LAItot during the times of LAI measurements), while shrubs dominated the high lawns and hummocks (58% ± 15% and 85% ± 15% of LAItot during the times of LAI measurements). Similar to the LAIaer of high lawn and hummock species, maximum LAIshrub was reached relatively late in the year, thereby supporting high LAItot values at the high lawns and hummocks into the fall.

#### 3.1.2 Variation in Pore Water Data

**FIGURE 4:**
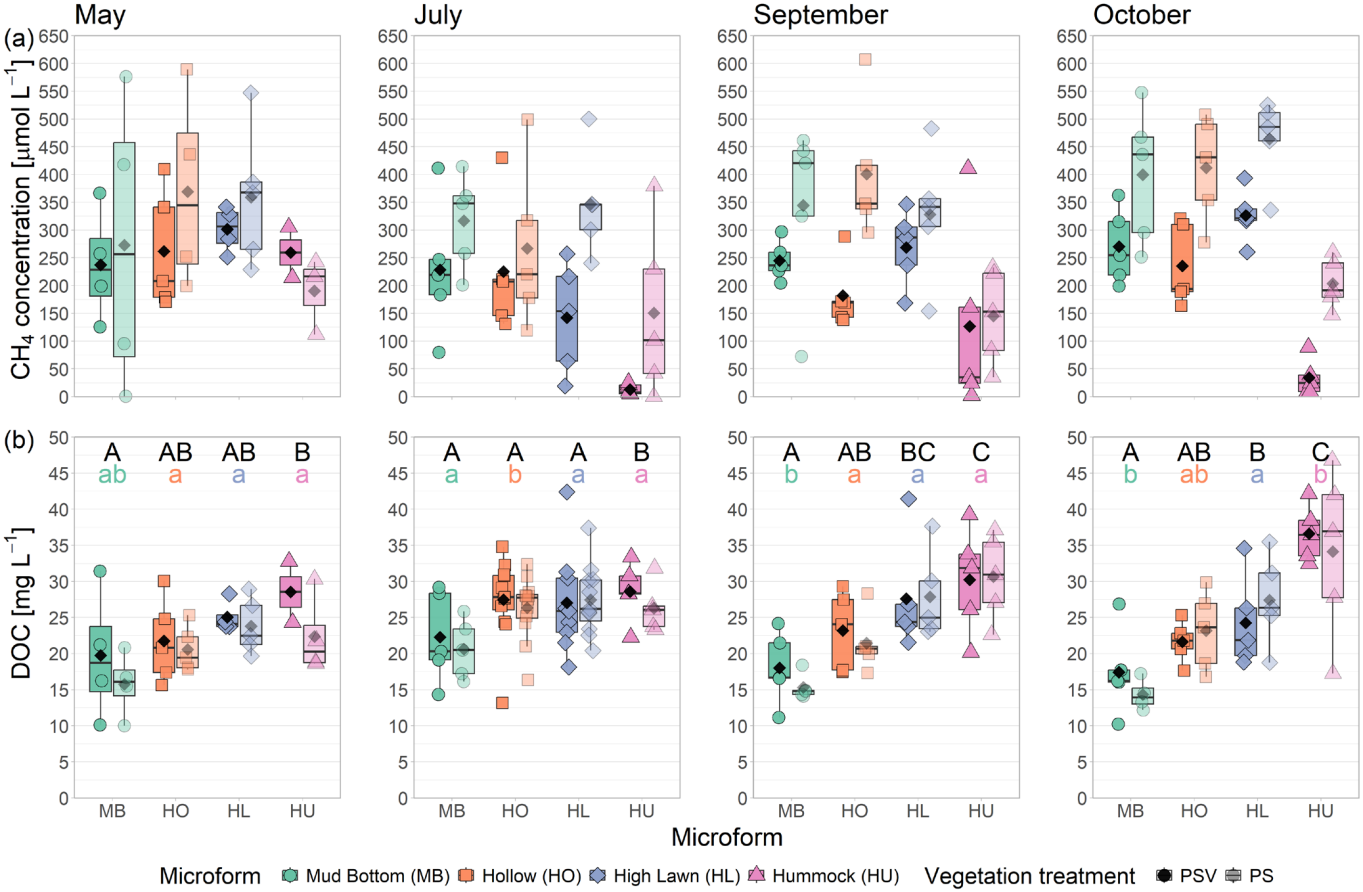
Concentrations of dissolved CH_4_ (a) and DOC (b) in the pore water at 20 cm depth by measurement campaign, microform and vegetation treatment. Markers show the individual values, the boxplot shows the median (horizontal line), 25th and 75th percentiles (hinges) and smallest/largest values, no more than 1.5 times the inter‐quartile range from the hinges (whiskers). Values above/below the whiskers are classified as outliers. Mean values are given as black diamonds. In (b), different capital letters indicate significant differences (*p* < 0.05) between microforms within one measurement campaign and different small, coloured letters indicate significant differences between measurement campaigns for one microform. Individual differences are not shown in (a), as measurement campaign, microform and vegetation treatment did not interact in their effects on pore water CH_4_ concentrations. In (b), treatment differences are not shown as the vegetation treatment did not significantly affect the DOC concentrations in the pore water.

Concentrations of CH_4_ dissolved in the pore water at 20 cm depth differed significantly between measurement campaigns (*F*_(3,112.39)_ = 4.166, *p* = 0.0077), microforms (*F*_(3,34.86)_ = 19.399, *p* < 0.0001) and vegetation treatments (*F*_(1,34.54)_ = 29.347, *p* < 0.0001). Pore water CH_4_ concentrations were significantly lower at the hummocks than at the other microforms, particularly when the water table at the hummocks dropped below the sampling depth between July and October. Furthermore, pore water concentrations were significantly lower at the control plots with intact vegetation (PSV) than at the plots where vascular plants had been removed (PS) and significantly lower in July than in May and October. Although no significant interaction was detected between the effects of the measurement campaign and vegetation treatment, visual inspection of the data suggests that treatment differences occurred in July, September and October but were non‐significant in May (Figure 4a).

Seasonal patterns in DOC concentrations at 20 cm depth differed significantly between microforms (campaign by microform interaction *F*_(9,126.17)_ = 4.974, *p* < 0.0001). At the wetter microforms (mud bottoms and hollows), DOC concentrations showed a seasonal cycle, increasing between spring and summer and then decreasing again towards the fall, while at the drier microforms (high lawns and hummocks), DOC concentrations remained constant over the study period and were even significantly higher at the hummocks in October compared to the other months (Figure 4b). The overall pattern of increasing DOC concentrations with increasing surface height of the microforms was therefore most pronounced in fall. Although DOC concentrations increased significantly with increasing LAItot (*F*_(1,93.99)_ = 28.221, *p* < 0.0001), they did not differ significantly between the vegetation treatments.

### 3.2 Variation in CH_4_ Fluxes

#### 3.2.1 CH_4_ Fluxes From Plots With Intact Vegetation

Mean CH_4_ fluxes at the plots with intact vegetation (PSV) ranged from 29 ± 8 mg CH_4_ m^−2^ day^−1^ at the hummocks in spring to 262 ± 194 mg CH_4_ m^−2^ day^−1^ at the hollows in summer. Net uptake of CH_4_ was recorded three times at hummocks in July (Figure 6). Seasonal variation in CH_4_ emissions from the PSV plots differed significantly between microforms (campaign by microform interaction: *F*_(9,168.91)_ = 3.048, *p* = 0.0021) depending on the hydrological conditions. Emissions from the wetter microforms (mud bottoms and hollows) showed a pronounced seasonal pattern, while emissions from the drier microforms (high lawns and hummocks) remained rather constant throughout the study period. A post hoc test based on the model considering the full data set, including all measurement campaigns, microforms and vegetation treatments, indicated significant seasonal variation only at the mud bottoms, featuring a significant decrease in mean CH_4_ emissions by 83% from May and July to October (Table S2). A decrease in mean CH_4_ emissions between summer and fall by 65% was visible also at the hollows and became statistically significant when only the hollow data was considered in the model (Table 1). At the high lawns and hummocks, on the contrary, both microform‐specific models and the full model suggested constant CH_4_ emissions from the PSV plots over all measurement campaigns of 167 ± 205 and 47 ± 88 mg CH_4_ m^−2^ day^−1^, respectively (high lawns: *F*_(3,42.13)_ = 0.702, *p* = 0.5559, hummocks: *F*_(3,51.65)_ = 1.121, *p* = 0.3493).

**TABLE 1:** Best multiple regression results for CH_4_ fluxes from the control plots with intact vegetation (PSV), the vegetation treatment with all vascular plants removed (PS) and the bare peat treatment (P) for all microforms and for each microform individually as well as for the calculated effects of vascular plants and of the *Sphagnum* moss layer on the CH_4_ fluxes.

Model	Microform	Coefficients	Estimate	SE	DF	*t* value	*p*	Marginal *R* ^2^	Conditional *R* ^2^
CH_4_ flux (PSV)	All	Intercept LAIaer	2.01 0.15	0.08 0.04	18.81 195.37	25.909 3.580	< 0.0001*** 0.0004***	0.06	0.30
	Mud bottom	Intercept LAIaer WTD	1.90 0.26 −0.20	0.09 0.07 0.08	3.39 36.12 29.70	20.993 4.032 −2.409	0.0001*** 0.0003*** 0.0224*	0.28	0.42
	Hollow	Intercept LAIaer	2.20 0.19	0.07 0.05	3.85 46.97	32.276 3.900	< 0.0001*** 0.0003***	0.25	0.35
	High lawn	Intercept	2.26	0.18	4.00	12.361	0.0002***	0	0.46
	Hummock	Intercept	1.70	0.16	3.97	10.508	0.0005***	0	0.12
CH_4_ flux (PS)	All	Intercept T20 WTD	1.34 0.18 −0.19	0.10 0.06 0.10	13.01 98.73 27.98	13.722 2.879 −1.904	< 0.0001*** 0.0049** 0.0672	0.08	0.31
	Hollow	Intercept T20 WTD	1.50 0.89 −0.74	0.10 0.14 0.15	3.27 37.22 25.85	14.932 6.494 −4.917	0.0004*** < 0.0001*** < 0.0001***	0.53	0.58
	High lawn	Intercept T20 WTD	1.11 0.51 −0.59	0.22 0.22 0.22	3.89 43.20 42.96	5.004 2.296 −2.647	0.0080** 0.0266* 0.0113*	0.12	0.49
	Hummock	Intercept	1.39	0.10	3.92	14.352	0.0002***	0	0.09
CH_4_ flux (P)	All	Intercept	1.72	0.14	18.94	11.928	< 0.0001***	0	0.55
	Mud bottom	Intercept	1.10	0.20	3.79	5.608	0.0058**	0	0.03
	Hollow	Intercept WTD	2.40 0.12	0.17 0.06	3.96 26.68	13.939 1.871	0.0002*** 0.0723	0.05	0.62
	High lawn	Intercept WTD	2.00 −0.20	0.18 0.09	3.92 28.62	10.880 −2.317	0.0004*** 0.0279*	0.10	0.46
	Hummock	Intercept	1.41	0.20	3.94	7.083	0.0022**	0	0.26
Vascular plant effect		Intercept LAIaer T20	1.92 0.32 −0.19	0.10 0.10 0.09	19.29 104.38 103.81	19.040 3.261 −2.090	< 0.0001*** 0.0015** 0.0390*	0.10	0.32
*Sphagnum* effect		Intercept T20 WTD T20: WTD	247.28 8.15 −72.95 −77.93	72.49 34.04 63.12 30.49	13.70 74.81 32.37 88.04	3.41 0.24 −1.16 −2.56	0.0043** 0.8115 0.2562 0.0123*	0.07	0.75

*Note:* As predictor variables, we considered peat temperature at 20 cm depth (T20), WTD, LAItot and LAIaer as well as their interactions. The predictor variables were standardized to account for the difference in their units and scales and a plot identifier was included in all models as a random effect. CH_4_ fluxes and vascular plant effects were pseudo‐log transformed to meet the assumption of normality.

* 0.01 < *p* < 0.05.

** 0.001 < *p* < 0.01.

*** 0 < *p* < 0.001.

Seasonally changing CH_4_ emissions from the wetter microforms, as opposed to constant CH_4_ emissions from the drier microforms, caused a shift in the spatial pattern in CH_4_ emissions between the measurement campaigns. In May and July, CH_4_ emissions were lowest at the hummocks, being 82% lower than mud bottom and high lawn emissions in May and 84% lower than hollow and high lawn emissions in July. By September, this pattern changed as mud‐bottom and hollow emissions decreased, while high‐lawn and hummock emissions remained stable. By October, mean emissions were 87% higher at the high lawns compared to the mud bottoms, and hummock emissions were similar to those of the other microforms.

The variation in CH_4_ fluxes at plots with intact vegetation was best explained by variations in LAIaer (Table 1, Figure S5). Although no significant interaction effect of LAIaer with WTD or microform was found, the effect of increasing CH_4_ emissions with increasing LAIaer was significant only at the wetter microforms (Table 1) but insignificant at the drier microforms (high lawns: *F*_(1,45.32)_ = 0.749, *p* = 0.3913; hummocks: *F*_(1,17.53)_ = 4.421, *p* = 0.0502), when a separate model was built for each microform. As suggested by the high spring emissions despite low LAIaer values (Figures 3d and 6), the amount of variance in the mud bottom and hollow emissions explained by LAIaer as a single predictor increased by 35% and 10%, respectively, when only the observations from summer and fall were considered. At the mud bottoms, CH_4_ emissions increased significantly with rising water table, dampening the effect of LAIaer on the CH_4_ emissions (Table 1).

**FIGURE 5:**
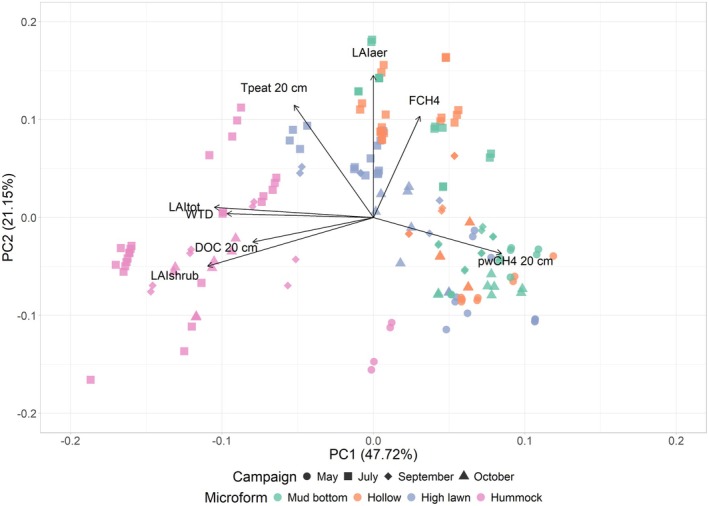
PCA ordination diagram for the measurement plots by measurement campaign (marker shape) and microform (marker colour). Black arrows indicate the direction and the strength of the effect of the LAI of all vascular plants (LAItot), of aerenchymatous species (LAIaer), and of shrubs (LAIshrub), the water table depth (WTD), the peat temperature at 20 cm depth (Tpeat 20 cm), the concentrations of dissolved CH_4_ (pwCH4 20 cm) and DOC in the pore water at 20 cm depth (DOC 20 cm), and the pseudo‐logarithmically transformed CH_4_ emissions (FCH_4_) on the spatial and seasonal variation between the measurements. Eigenvalues for axis 1 and 2 are 4.010 and 1.778, respectively, and together, axes 1 and 2 explain 69% of the variation in the data.

The hummock plots differed from the other microforms in all studied seasons, while conditions at the mud bottoms, hollows and high lawns were similar in the shoulder seasons, and high lawns differed from mud bottoms and hollows only in summer, as indicated by our PCA analysis (Figure 5). Principal components (PC) 1 and 2 explained 52% and 19% of the variation in the data, respectively. The microforms differed mostly from each other along PC 1, which was negatively correlated with LAItot, LAIshrub, WTD and DOC at 20 cm depth with correlation coefficients (*r*) of −0.5, −0.5, −0.4 and −0.4, respectively, and positively with pore water concentrations of dissolved CH_4_ at 20 cm depth (*r* = 0.4). CH_4_ emissions, peat temperatures at 20 cm depth, and LAIaer showed a positive correlation with PC 2 (*r* = 0.5, 0.5 and 0.7, respectively), along which most of the seasonal variation in the measurements occurred.

#### 3.2.2 CH_4_ Fluxes From the Vegetation Removal Treatments

The magnitude of CH_4_ fluxes as well as their seasonal and spatial patterns changed when the vascular plants and the *Sphagnum* moss layer were removed from the measurement plots (Figure 5, Figure S6; campaign by microform by treatment interaction: *F*_(15,390.75)_ = 2.331, *p* = 0.0034). Overall, removing the vascular plants led to a decrease in CH_4_ emissions, while also removing the moss layer increased the CH_4_ emissions again to levels similar to the intact vegetation. Differences between the vegetation treatments were lowest in spring of all seasons and at the hummocks of all microforms.

**FIGURE 6:**
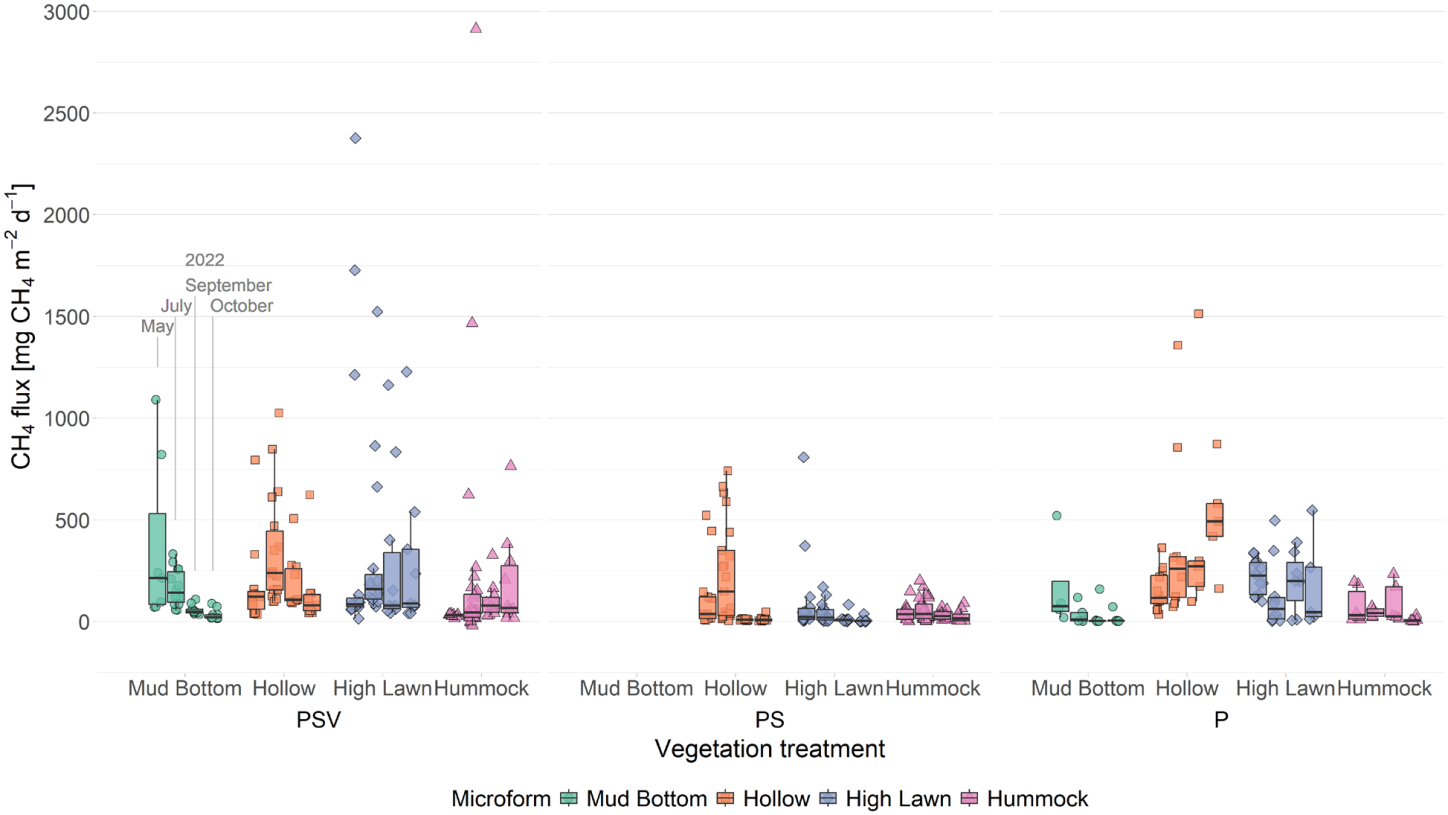
Seasonal variability in CH_4_ fluxes by vegetation treatment (control plots with intact vegetation [PSV], moss‐only plots [PS], bare beat plots [P]) and microform. Flux values are split into the measurement campaigns of May, July, September and October 2022. Markers show the individual values, the boxplot shows the median (horizontal line), 25th and 75th percentiles (hinges) and smallest/largest values, no more than 1.5 times the inter‐quartile range from the hinges (whiskers). Values above/below the whiskers are classified as outliers. Significant differences between measurement campaigns, microforms and vegetation treatments are listed in Table S2.

**FIGURE 7:**
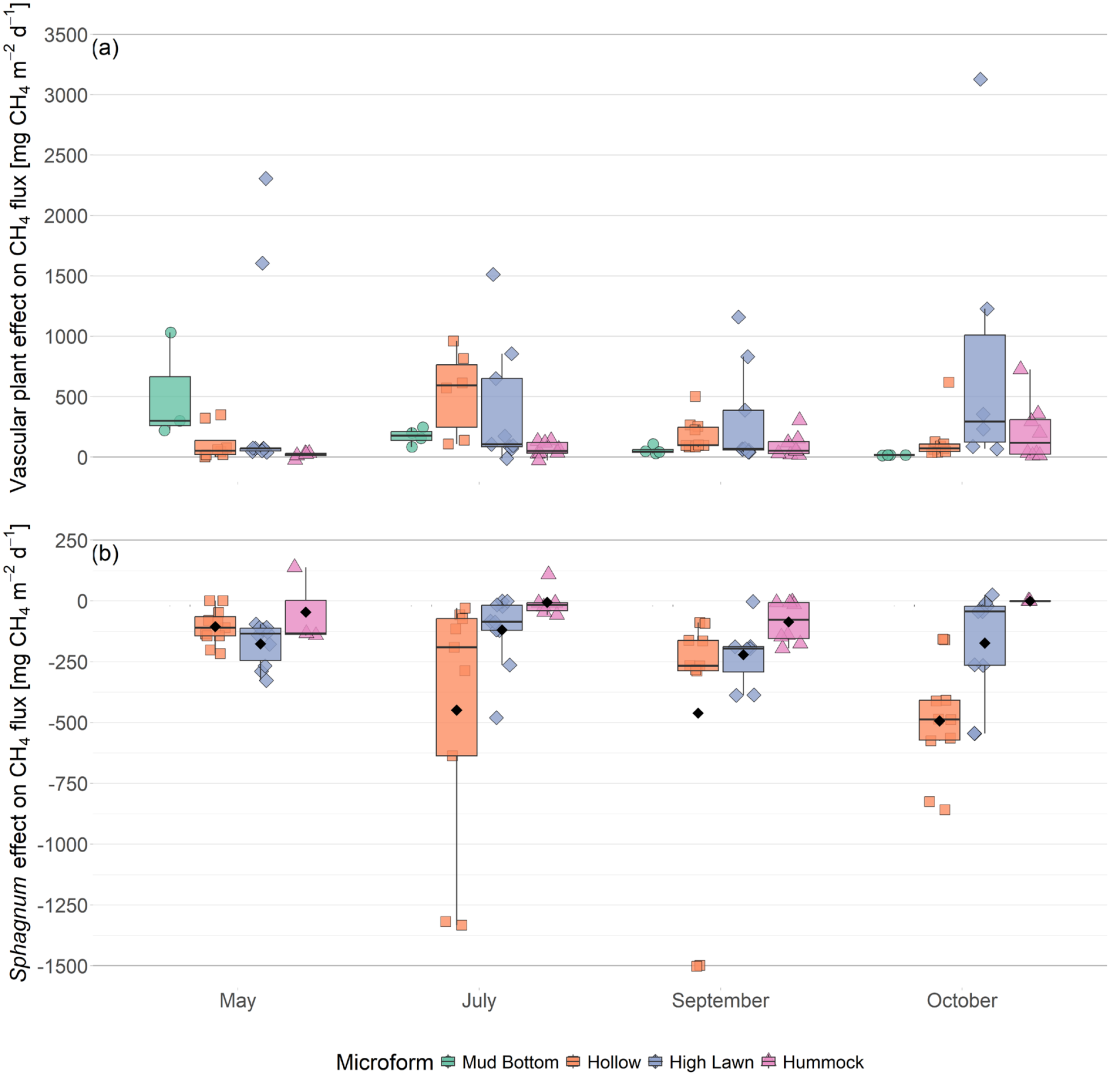
Effects of vascular plants (a), and the *Sphagnum* moss layer (b) on the CH_4_ fluxes by microform and measurement campaign. Positive values indicate an increasing effect of vegetation on the CH_4_ emissions. Markers show the individual values, the boxplot shows the median (horizontal line), 25th and 75th percentiles (hinges) and smallest/largest values, no more than 1.5 times the inter‐quartile range from the hinges (whiskers). Values above/below the whiskers are classified as outliers. In (b), mean values are given as black diamonds. Mean values are not shown in (a) because vascular plant effects were not normally distributed. Significant differences between measurement campaigns and microforms are listed in Table S3.

Removing vascular plants (PS plots) significantly reduced CH_4_ emissions from hollows and high lawns, especially in fall. This intensified the observed decrease in hollow emissions from July to October at the PSV plots to 92% and introduced a significant 86% decrease in high lawn emissions. As a result, fall emissions from both microforms became significantly lower than their spring emissions. Similar to the PSV plots, fall emissions from the mud bottoms were significantly lower by 93% than the respective spring emissions also when vascular plants were removed, while the summer emissions showed large variation in the absence of vascular plants and therefore did not differ significantly from the other seasons. Hummock emissions were least affected by the removal of the vascular plants and, similar to the PSV plots, remained constant over the study period at a slightly lower level of 25 ± 30 mg CH_4_ m^−2^ day^−1^ when the vascular plants were removed. As the spatial pattern in vascular plant effects resembled the spatial pattern in CH_4_ emissions from the PSV plots (Figures 6 and 7a), significant spatial differences in CH_4_ emissions disappeared once the vascular plants were removed (Table S2). Due to the relatively low vascular plant effect at the hummocks, mean CH_4_ emissions, particularly from the high lawns, even dropped below the hummock emissions at the PS treatment. In the absence of vascular plants, the seasonal pattern of CH_4_ emissions—typically higher in summer than in the shoulder seasons—was best explained by an increase in CH_4_ emissions with increasing peat temperature at 20 cm depth (Table 1, Figure S7). At the hollows and high lawns, a significant increase in CH_4_ emissions with rising water table counteracted the temperature effect (Table 1). This WTD dependency became insignificant, however, when excluding the spring observations from the models (hollows: *F*_(1,7.55)_ = 2.896, *p* = 0.1294; high lawns: *F*_(1,29.94)_ = 0.003, *p* = 0.9544). Peat temperatures did not have a significant effect on hummock emissions (*F*_(1,51.44)_ = 0.199, *p* = 0.6578).

Once the moss layer was removed as well (P plots), spring CH_4_ emissions from the high lawns became significantly larger than their summer emissions, while any other seasonal variation disappeared at the hollows and high lawns. At the hummocks, on the other hand, mean CH_4_ emissions in October were 88% lower than earlier in the year. When the moss layer was removed, CH_4_ emissions strongly increased at the hollows and high lawns, especially during fall. This made their emissions significantly higher compared to the low emissions from the naturally moss‐free mud bottoms. As hollow and high lawn emissions showed no seasonality at the P plots, the spatial pattern of decreasing CH_4_ emissions from hollows to high lawns to hummocks, similar to the PSV plots in summer, remained consistent at the P plots between July and October. While none of the considered environmental variables showed a significant effect on the CH_4_ emissions from the P plots when pooling all microforms together, CH_4_ emissions from the high lawns increased significantly with a rising water table (Table 1, Figure S8).

The share of measurements showing one or more ebullition events differed between measurement campaigns and vegetation treatments. The highest share of measurements showing ebullition events was detected in summer across all microforms and vegetation treatments (Figure S2). Ebullition occurred more frequently at wetter than at drier microforms, with the share of measurements showing ebullition events being highest at the mud bottoms and lowest at the hummocks across all seasons, particularly at the intact vegetation plots (Table 2). Of all vegetation treatments, measurements at the bare peat plots showed the highest number of ebullition events.

**TABLE 2:** Absolute and relative areal contributions of landcover classes at Siikaneva bog.

Landcover class	Area [ha]	Areal contribution to Siikaneva bog [%]	Share of chamber measurements with ebullition events [%]
Open water	2.935 ± 0.228	3.93 ± 0.31	
Mud bottom	**3.587 ± 0.638**	**4.81 ± 0.86**	**48**
Hollow	**21.578 ± 7.371**	**28.91 ± 9.87**	**39**
Lawn*	9.527 ± 2.660	12.76 ± 3.56	
High lawn	**20.972 ± 5.304**	**28.10 ± 7.11**	**35**
Hummock	**14.189 ± 5.376**	**19.01 ± 7.20**	**27**
Mineral soil island	1.656 ± 0.204	2.22 ± 0.27	
Boardwalk	0.201 ± 0.025	0.27 ± 0.03	
Total	74.645	100	

*Note:* Areal contributions of landcover classes that were studied at the plot level are marked in bold. The last column gives the percentage of chamber measurements at the control (PSV) plots that showed one or more ebullition events.

* No chamber measurements were performed at the lawns, but they were considered in the upscaling to the ecosystem scale.

### 3.3 Variation in Vegetation Effects on CH_4_ Fluxes

Vascular plants enhanced the CH_4_ emissions in all seasons and at all microforms (Figure 7a). The enhancement by vascular plants increased significantly with increasing LAIaer (Table 1). Similar to LAIaer, the mean vascular plant effect decreased between July and October at the wetter microforms, while at the drier microforms, the vascular plant effect remained constant even when their LAIaer dropped significantly between September and October. The spatial pattern in vascular plant effects therefore gradually shifted from higher effects at the wetter microforms in July towards higher effects at the drier microforms in October. Similar to LAIaer, the vascular plant effect at the hummocks was significantly lower in spring than in the other seasons (Table S3). Overall, the vascular plant effect was high in spring, particularly at the mud bottoms, considering the LAIaer close to zero at all microforms. Multiple linear regression similarly suggested an increase in vascular plant effect with decreasing peat temperatures (Table 1).

On average, CH_4_ emissions decreased in the presence of *Sphagnum* moss (Figure 7b). In the hollows, the mean effect of the *Sphagnum* layer on CH_4_ emissions in fall (478 ± 413 mg CH_4_ m^−2^ day^−1^) was nearly five times as high as in spring (106 ± 73 mg CH_4_ m^−2^ day^−1^). At the high lawns and hummocks, the *Sphagnum* effect did not vary significantly between the seasons, on average decreasing the CH_4_ emissions by 169 ± 154 and 46 ± 90 mg CH_4_ m^−2^ day^−1^ across all measurement campaigns, respectively. At the high lawns, the *Sphagnum* effect still visibly varied between the seasons, significantly affecting the CH_4_ emissions during the shoulder seasons but not in summer, while at the hummocks, the *Sphagnum* effect remained lowest and insignificant year‐round. Spatial differences in the *Sphagnum* effect were not significant in any season, but a pattern of decreasing mean values from hollows to high lawns to hummocks was found between July and October. In spring, the mean *Sphagnum* effect was highest at the high lawns and insignificant at the other microforms. This seasonal change in the spatial pattern is reflected in the interacting effects of WTD and peat temperature on the *Sphagnum* effect (Table 1).

### 3.4 Areal Contribution of Land Cover Classes at Siikaneva Bog

The largest parts of Siikaneva bog are covered by hollows and high lawns, which together account for 57% of the bog area (Table 2). Of the microforms considered in this study, mud bottoms contributed least to the area of Siikaneva bog. The microforms that were represented in the chamber measurements account for 80.8% of the area or 93.6% when lawns are considered as well.

### 3.5 Upscaled CH_4_ Fluxes

**FIGURE 8:**
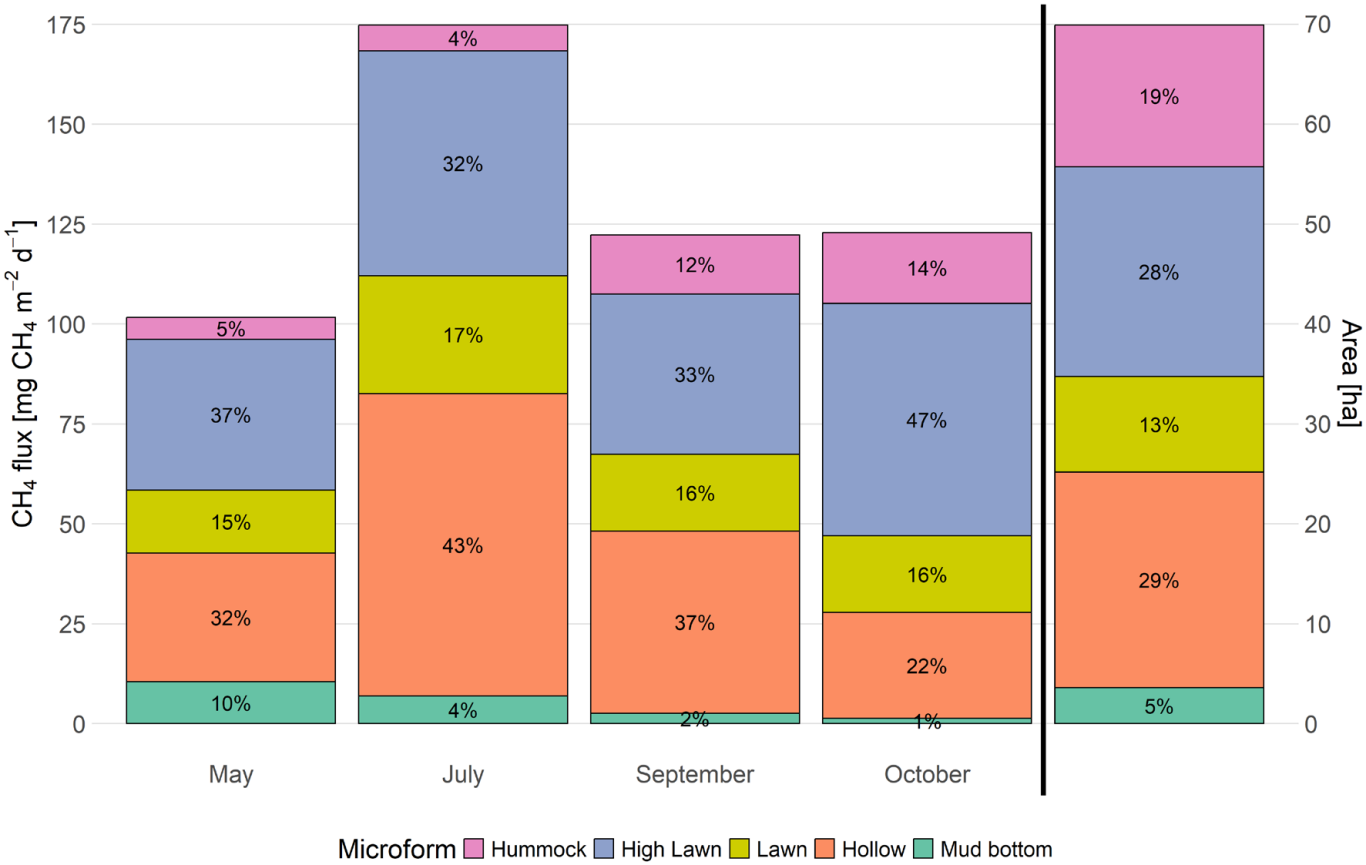
Upscaled CH_4_ emissions from Siikaneva bog by season and microform (bars 1–4, left *y*‐axis), compared to the relative areal contribution of each microform (bar 5, right *y*‐axis). The seasonal emissions are shown as stacked bar graphs, with each colour representing a different microform (mud bottom, hollow, lawn, high lawn, hummock). The fifth bar, separated by a black vertical line, shows the proportional bog area covered by the studied microforms (69.853 ha in total).

Spatially upscaled CH_4_ fluxes from Siikaneva bog were highest in summer and lower during the shoulder seasons, with the spring emissions being 42% lower than the summer emissions and September and October emissions being 30% lower, respectively (Figure 8, Table S4). Matching their low areal coverage, the contributions of mud bottoms, lawns and hummocks to the CH_4_ emissions were lower in all seasons than the contributions of hollows and high lawns, which cover larger areas of the bog (Table 2, Figure 8). The wet hollows clearly dominated the bog emissions in summer. While in spring the contribution of hollows to the CH_4_ emissions matched their areal contribution to the bog area, their summer contribution to the emissions was 1.5 times as high as their areal contribution. Between July and October, the contribution of wetter microforms (mud bottoms and hollows) to the bog emissions decreased, while the contribution of intermediate lawns remained constant, and emissions from drier microforms (high lawns and hummocks) gained relative importance in their contribution to the total CH_4_ emissions from Siikaneva bog. Depending on the season, mud bottoms and hollows contributed more or less strongly to the emissions than suggested by their areal contributions. Lawns and high lawns, on the contrary, emitted more CH_4_, while hummocks emitted less CH_4_ in all seasons than expected based on their area.

## 4 Discussion

Seasonal patterns in CH_4_ fluxes varied spatially along the microtopographic gradient of Siikaneva bog. At the wetter microforms (mud bottoms and hollows), emissions followed the seasonal cycle that is typically associated with the temperature dependency of CH_4_ production, whereas drier microforms (high lawns and hummocks) showed seasonally rather constant CH_4_ emissions (Figure 6). Upscaling the microtopographical‐scale flux measurements, we found that areal contributions of drier microforms can therefore reduce seasonality in ecosystem‐scale CH_4_ emissions (Figure 8, Table S4). Neglecting microtopographical differences when upscaling in situ measurements or in process‐based modelling can therefore introduce a seasonally changing bias into estimates of ecosystem‐scale emissions, including an underestimation of CH_4_ emissions outside the peak growing season. Understanding the environmental conditions that promote seasonally constant CH_4_ emissions is essential for evaluating how microtopographical differences influence both the seasonal variability in ecosystem‐scale emissions and potential future changes in CH_4_ emissions from patterned peatlands.

### 4.1 Drivers of Seasonal Patterns in CH_4_ Emissions Across Microforms

#### 4.1.1 Seasonally Constant CH_4_ Emissions From Drier Microforms

We identified several reasons for seasonally stable CH_4_ emissions related to a low water table and its direct and indirect effects on CH_4_ production, oxidation and transport. Specifically, constant or even increasing CH_4_ emissions from drier microforms between summer and fall were caused by a combination of (1) a reduced temperature dependency of CH_4_ production, (2) missing WTD limitation of CH_4_ oxidation and (3) a seasonally stable effect of vascular plants on CH_4_ emissions.

1. A reduced temperature dependency of CH_4_ production at low water levels in our study was caused both by a lower peat temperature effect on CH_4_ production and lower seasonal variability in peat temperatures at depths of CH_4_ production in the peat profile.

In the presence of vascular plants, variability in CH_4_ emissions was best explained by variations in LAIaer (Table 1). However, an underlying temperature dependency of CH_4_ production at all microforms (Dunfield et al. 1993) is still suggested by a high correlation between LAIaer and peat temperature, a positive association of CH_4_ emissions with peat temperature in vascular plant removal treatments (Table 1), and low hummock emissions from bare peat treatments in fall (Figure 6). The temperature dependency of CH_4_ emissions decreased, however, with increasing WTD (Table 1). Such lower temperature dependency of CH_4_ emissions at lower water tables has previously been found by Svensson and Rosswall (1984), Nykänen et al. (1998) and Frenzel and Karofeld (2000).

Additionally, at the drier microforms, CH_4_ production was confined to deeper peat layers that were less exposed to air temperature changes than the surface zone where CH_4_ oxidation occurs (Figure S1). Due to the stronger temperature fluctuations in the surface zone, at low water tables, the temperature effect on CH_4_ oxidation might compensate for the generally higher temperature dependency of CH_4_ production (Kutzbach et al. 2004), resulting in constant CH_4_ emissions between summer and fall despite strongly decreasing air temperatures (Figures 3b and 6).

2 Seasonal variability in water table depth had no effect on CH_4_ emissions from drier microforms as it remained both below a level at which it would restrict oxidation rates as well as below the living *Sphagnum* layer throughout our study period (Figure 3a).

A pronounced decrease in CH_4_ emissions associated with the *Sphagnum* layer (Figure 7b) indicates highly efficient CH_4_ oxidation occurring within the moss layer. This oxidation appears to cease only when the water table rises above the moss surface, as evidenced by the low *Sphagnum* effect and high CH_4_ emissions from hollows in spring (Figure 6). This observation is supported by earlier findings that highlight a symbiotic relationship between *Sphagnum* mosses and methanotrophic bacteria, which enables highly efficient CH_4_ oxidation (Kip et al. 2010; Larmola et al. 2010).

Within the *Sphagnum* layer, oxidation was limited mainly by the availability of CH_4_ to be oxidized (Jentzsch et al. 2024a). CH_4_ concentrations in the *Sphagnum* layer, in turn, were controlled partly by WTD as the potential for CH_4_ oxidation is highest close to the water table (Frenzel and Karofeld 2000). This is indicated by a lower effect of the *Sphagnum* layer on CH_4_ emissions at lower water tables across all microforms with a naturally occurring moss layer (Table 1; Larmola et al. 2010). Low pore water CH_4_ concentrations associated with efficient oxidation in deeper peat layers, therefore, prevented significant CH_4_ oxidation in the moss layer of hummocks year‐round (Figure 7b), particularly when the water table dropped below the sampling depth between July and October (Figure 4a).

3 The different seasonal behavior of the vascular plant effect between wetter and drier microforms can be explained by differences in the composition of the vascular plant community, regarding both the individual plant species as well as plant functional types (PFTs) (Table S1).

The presence of vascular plants generally enhanced CH_4_ emissions at all microforms (Figure 7a). Seasonal and spatial variability in the enhancing effect of vascular plants was best described by variations in LAIaer (Table 1). Due to the high magnitude of the vascular plant effect, variations in LAIaer also introduced most of the seasonality in net CH_4_ emissions across all microforms (Table 1). Seasonality in LAIaer differed between the microforms, however, thereby introducing different seasonality in vascular plant effects and consequently in net CH_4_ emissions depending on the microform. Peak LAIaer at high lawns and hummocks was reached approximately 1 month later (end of August/beginning of September) than at the mud bottoms and hollows (end of July/beginning of August) (Figure S4). Differences in the growing cycle have also been found at the species level: the growing cycle of *E. vaginatum*, the dominant sedge species at high lawns and hummocks, is delayed compared to those of *R. alba* and *S. palustris* , which dominate mud bottoms and hollows, respectively (Korrensalo et al. 2022). Species differences in growing cycles likely result from the combined effects of species characteristics and habitats. Lower peat temperatures at the drier microforms in spring (Figure 3a) lead to a retention of ice lenses in hummocks well into the growing season (personal observation and Nungesser 2003) and likely delayed the onset of plant growth.

We have previously shown that the increasing effect of vascular plants on hollow emissions is caused mainly by effective CH_4_ transport through aerenchymatous plants, which is strongly, although not exclusively, controlled by LAIaer (Jentzsch et al. 2024a; Korrensalo et al. 2022). Plant‐mediated transport was the dominating effect of vascular plants also at the other microforms, as the presence of vascular plants consistently reduced pore water CH_4_ concentrations (Figure 4a) while enhancing net CH_4_ emissions (Figure 7a). However, *E. vaginatum* , the dominant aerenchymatous plant species at the high lawns and hummocks, has been shown to transport CH_4_ at significantly lower rates than *R. alba* and *S. palustris* , which dominate the mud bottoms and hollows, respectively (Korrensalo et al. 2022). This might explain why variations in LAIaer significantly affected CH_4_ emissions only at the mud bottoms and hollows but not at the high lawns and hummocks when considering the microforms separately (Table 1).

Instead, part of the enhancing effect of vascular plants might be related to substrate supply from plant litter which is not directly correlated with momentary LAIaer (Li et al. 2010). At the high lawns and hummocks, shrubs contributed significantly to the vascular plant layer (58% and 85% of LAItot), resulting in a significantly higher LAItot than at the mud bottoms and hollows, where the vascular plant community consisted almost exclusively of aerenchymatous plants (98% and 88% of LAItot) (Figure 3c). The positive correlation between LAItot and DOC concentrations in the pore water (Figure 5) suggests that the vascular plants provide organic material which can potentially serve as substrate for CH_4_ production, as explained by Joabsson et al. (1999). Strongly increasing DOC concentrations with increasing surface height of the microforms in fall and higher DOC concentrations at the hummocks in fall than in summer (Figure 4b), despite slightly decreasing LAItot (Figure 3c), indicate that plant decay towards the end of the growing season led to additional input of organic material in fall. However, momentary DOC concentrations in the pore water cannot be directly related to substrate supply for methanogenesis, as they are a result of both momentary and past provision and turnover of DOC. Furthermore, only a fraction of DOC like organic acids such as acetate can serve as substrate for CH_4_ production and thus enhance CH_4_ emissions (Ström et al. 2003), while total DOC was previously found to be negatively correlated with CH_4_ production (Ström et al. 2003).

In addition to supplying substrates for methanogenesis, woody species such as dwarf shrubs and tree seedlings have been reported to contribute to CH_4_ emissions through transport from subsurface production zones and through in‐stem CH_4_ production (Halmeenmäki et al. 2017). While these CH_4_ transport rates are small compared to those in herbaceous wetland species (Ge et al. 2023, 2025), the role of woody vegetation in peatland CH_4_ dynamics may still warrant further attention, particularly given their different phenology and seasonal activity patterns. The predominance of evergreen species in the high lawns and hummocks of Siikaneva bog (Table S1) suggests the potential for year‐round, albeit modest, influence on CH_4_ fluxes.

#### 4.1.2 High Spring CH_4_ Emissions

Spring emissions were unexpectedly high across all microforms and vegetation treatments, despite low peat temperatures in the CH_4_ production zone and near‐zero LAIaer (Figures 3b,d and 6). These elevated fluxes, under environmental conditions that typically constrain both CH_4_ production and plant‐mediated transport, suggest that spring emissions are not solely driven by instantaneous microbial activity. Instead, a substantial portion of the emitted CH_4_ was likely produced over the winter and accumulated in the peat profile, with emissions suppressed by snow cover and a frozen surface layer. Upon thaw, this stored CH_4_ was rapidly released, contributing to the observed emission peak (Alm et al. 1999; Friborg et al. 1997; Tokida et al. 2007; Zona et al. 2016). This interpretation is supported by the relatively high concentrations of dissolved CH_4_ measured in the pore water in spring (Figure 4a). Moreover, the absence of clear differences in pore water CH_4_ concentrations between control and vegetation removal plots suggests that plant‐mediated transport was minimal during this period, potentially enhancing CH_4_ buildup in the peat and leading to elevated diffusive emissions (Figure 4a).

Although spring CH_4_ emissions were elevated across all microforms, they were less pronounced at drier microforms compared to wetter ones (Figure 6). This pattern likely reflects the influence of both lower water tables and lower peat temperatures at drier sites (Figure 3a,b). In wet microforms, high water tables near the surface inhibit CH_4_ oxidation, allowing a greater proportion of stored CH_4_ to be emitted upon thaw (Figure 7b). In contrast, drier microforms maintain water tables below the surface, facilitating CH_4_ oxidation. Additionally, lower peat temperatures at these sites reduce CH_4_ production (Frenzel and Karofeld 2000; Waddington and Roulet 1996) and delay snowmelt and surface thaw (Nungesser 2003), thereby postponing the release of CH_4_ produced over the winter.

#### 4.1.3 A Special Role of Mud Bottoms?

CH_4_ emissions from mud bottoms were surprisingly low compared to the drier microforms in summer and fall, particularly when all vegetation was removed (Figure 6) considering their high water table throughout the year (Figure 3a). This contradicts Karofeld (2004), who assumed high CH_4_ emissions from mud bottoms following the degradation of the moss layer and a consequent decrease in peat accumulation. As possible explanations for the low mud bottom emissions, we suggest low CH_4_ production rates (1), high rates of CH_4_ oxidation despite the high water table (2), and significant ebullitive CH_4_ emissions (3).

1. Low substrate availability might have limited CH_4_ production at the mud bottoms. Even when the vascular plants were removed, differences remained between the microforms in the availability of organic material in the peat, as indicated by the increase in DOC concentrations with increasing surface height (Figure 4b). Furthermore, the input of organic material from the *Sphagnum* layer might have still affected the CH_4_ production at the hollows, high lawns and hummocks, as it was removed only for the time of the chamber measurements, while at the mud bottoms the moss layer was naturally missing.
2. Furthermore, highly efficient CH_4_ oxidation can occur even at mud bottoms within an oxidizing layer just a few millimetres thick, as demonstrated by Frenzel and Karofeld (2000), who reported low CH_4_ emissions from mud bottoms despite the absence of an obvious oxidizing surface layer. This suggests that oxidation may still take place under water‐saturated conditions, where dissolved oxygen in the water can support CH_4_ oxidation. Given the slow diffusion rate of CH_4_ through water, even a thin oxidizing layer may be sufficient to effectively limit CH_4_ emissions. Measurements of surface oxygen concentrations could help confirm this mechanism.
3. Another explanation for low diffusive CH_4_ emissions from the mud bottoms could be a higher importance of ebullition compared to the other microforms. In the presence of vascular plants, ebullition events occurred more frequently at the mud bottoms than at the other microforms in all seasons (Figure S2). When all vegetation was removed, disabling plant‐mediated CH_4_ transport, more ebullition was observed from drier microforms than from the mud bottoms in spring and fall. However, ebullition from the hollows, high lawns and hummocks might have been triggered more strongly by the vegetation removal setup as the moss layer was removed and the collar was inserted into the peat only shortly before the start of the chamber measurement. A high importance of ebullition at the mud bottoms could also explain the apparent mismatch between low diffusive CH_4_ emissions and relatively high concentrations of CH_4_ dissolved in the pore water (Figure 4a).

### 4.2 Implications of Seasonal and Spatial Patterns for Current and Future Ecosystem‐Scale Emissions

The pronounced seasonal and spatial variability in CH_4_ emissions across microforms has important implications for estimating ecosystem‐scale CH_4_ fluxes from boreal peatlands. High fall emissions from dry microforms and elevated spring emissions from wet microforms may help explain the frequently observed discrepancy between measured and modelled cold‐season CH_4_ emissions at the ecosystem scale (Ito et al. 2023; Treat et al. 2018). Furthermore, the finding of high fall emissions from dry microforms challenges the common perception of hollows as year‐round hot spots for CH_4_ emissions (Cresto Aleina et al. 2016). It suggests that the understanding of hollows as the most important feature in terms of CH_4_ emissions from patterned bogs may be heavily influenced by the strong bias towards summer measurements (e.g., Bubier et al. 1995; Frenzel and Karofeld 2000; Macdonald et al. 1998; Waddington and Roulet 1996). These findings underscore the critical importance of accounting for microtopographic differences when estimating annual ecosystem‐scale CH_4_ budgets.

Microtopographic differences in CH_4_ emissions on the plot scale suggest that both the magnitude and the seasonality in ecosystem‐scale CH_4_ emissions depend on the areal contributions of the different microforms. At theSiikaneva bog, the contributions of wet and dry microforms to the total surface area were relatively balanced, resulting in ecosystem‐scale emissions close to the simple mean across all microforms (Table 2, Figure 8). However, in other patterned bogs, the distribution of microforms can vary widely, potentially leading to different emission dynamics. Accurate assessment of ecosystem‐scale CH_4_ budgets therefore requires not only microform‐specific flux data but also spatially explicit information on microtopographic composition and associated environmental conditions. Incorporating these microscale patterns and their seasonal dynamics into process‐based CH_4_ models is essential for improving predictions of peatland CH_4_ emissions under current and future climate conditions.

Accounting for microtopography is essential for predicting the response of peatland CH_4_ emissions to climate change. The microscale spatial diversity in environmental conditions results in seasonally more stable emissions at the ecosystem scale compared to emissions from individual wet microforms (Figures 6 and 8). This suggests that spatial heterogeneity may buffer the impact of environmental changes on CH_4_ fluxes also on longer time scales. Model simulations support this, showing that microtopographic variation enhances ecosystem resilience to environmental perturbations both by supporting a diversity of plant species and PFTs and through variability in the physical properties of microforms (Turetsky et al. 2012). Patterned peatlands are likely to retain wetter conditions even under declining water tables, as microtopography reduces runoff and promotes water retention (Cresto Aleina et al. 2015). Feedbacks between moisture conditions, unique characteristics of *Sphagnum* species and decomposition rates result in a long‐term stability of microtopographical structures (Nungesser 2003). While hummocks may become drier, wetter microforms may retain or even increase their moisture levels (Strack et al. 2008). As a result, potential declines in CH_4_ emissions from drier features may be compensated by stable or increasing emissions from wetter ones. Neglecting microtopography in process‐based models may therefore lead to an overestimation of drying impacts and an underestimation of future CH_4_ emissions from peatlands.

An important uncertainty remains regarding the fate of wet microforms—particularly hollows—under prolonged drought. If water tables drop below a critical threshold, drought‐sensitive hollow mosses may die off, potentially leading to the development of moss‐free mud bottoms (Karofeld et al. 2015). Alternatively, drier conditions may promote the establishment of more competitive hummock plant species. While both mud bottoms and hummocks tend to emit relatively low amounts of CH_4_ (Figure 6), they differ in their seasonal emission patterns and carbon uptake potential. Mud bottoms likely sequester less carbon dioxide (CO_2_) due to their lower standing biomass and productivity compared to hummocks (Figure S7; Korrensalo, Kettunen, et al. 2018). Thus, shifts in microtopography composition could significantly affect not only CH_4_ dynamics but also the broader carbon balance of peatland ecosystems. Together, these findings underscore the need to integrate microscale spatial variability into long‐term modelling and monitoring frameworks to more accurately assess peatland responses to climate change and project future greenhouse gas fluxes.

## 5 Conclusions

We evaluated the importance of capturing microscale spatial heterogeneity within a patterned bog to better understand the seasonal dynamics in ecosystem‐scale CH_4_ emissions. Specifically, we investigated the spatial and seasonal variability of CH_4_ fluxes across distinct microforms and related these patterns to changes in environmental and ecological conditions.

Hydrological variability across microforms significantly influenced the seasonal emission dynamics at Siikaneva bog. CH_4_ emissions from wetter microforms such as mud bottoms and hollows declined from summer to fall, following reductions in peat temperature and LAIaer. In contrast, emissions from drier high lawns and hummocks remained relatively stable year‐round, likely due to persistently low water tables that supported steady CH_4_ production and oxidation. This temporal stability was further reinforced by the presence of evergreen shrubs and late‐growing sedge species, which sustained plant‐driven CH_4_ fluxes beyond the summer peak in air and peat temperatures.

Spring emissions were unexpectedly high, especially at wet microforms where elevated peat temperatures and saturated conditions favoured early CH_4_ production and suppressed oxidation. Additionally, the release of CH_4_ accumulated beneath a frozen surface layer over winter likely contributed to this spring peak. These findings suggest that microtopographic differences in the seasonality of CH_4_ fluxes may help explain higher‐than‐expected emissions outside the peak growing season.

The strong spatial heterogeneity in CH_4_ emissions and its seasonal variation underscore the sensitivity of both the magnitude and timing of ecosystem‐scale CH_4_ fluxes to the relative areal contributions of different microforms. Accurately quantifying and incorporating this microscale spatial structure is therefore critical for improving CH_4_ budget estimates and modelling efforts. Neglecting microtopographic variability in models may lead to overestimation of drying impacts and underestimation of future CH_4_ emissions, especially as climate change alters precipitation regimes and snow cover dynamics in boreal regions.

## Author Contributions

**Katharina Jentzsch:** conceptualization, data curation, formal analysis, investigation, methodology, software, validation, visualization, writing – original draft, writing – review and editing. **Elisa Männistö:** conceptualization, data curation, methodology, writing – review and editing. **Maija E. Marushchak:** conceptualization, data curation, methodology, software, supervision, writing – review and editing. **Tabea Rettelbach:** methodology, visualization, writing – original draft, writing – review and editing. **Lion Golde:** data curation, methodology. **Aino Korrensalo:** conceptualization, methodology, supervision, writing – review and editing. **Joshua Hashemi:** methodology. **Lona van Delden:** conceptualization, methodology. **Eeva‐Stiina Tuittila:** conceptualization, resources, supervision. **Christian Knoblauch:** supervision, writing – review and editing. **Claire C. Treat:** conceptualization, funding acquisition, project administration, supervision, writing – review and editing.

## Conflicts of Interest

The authors declare no conflicts of interest.

## Supporting information


Data S1.

## Data Availability

The data that support the findings of this study are openly available in PANGAEA at https://doi.org/10.1594/PANGAEA.971358.

## References

[gcb70372-bib-0001] Albuhaisi, Y. A. Y. , Y. Van Der Velde , and S. Houweling . 2023. “The Importance of Spatial Resolution in the Modeling of Methane Emissions From Natural Wetlands.” Remote Sensing 15, no. 11: 2840. 10.3390/rs15112840.

[gcb70372-bib-0002] Alekseychik, P. , A. Korrensalo , I. Mammarella , et al. 2021. “Carbon Balance of a Finnish Bog: Temporal Variability and Limiting Factors Based on 6 Years of Eddy‐Covariance Data.” Biogeosciences 18, no. 16: 4681–4704. 10.5194/bg-18-4681-2021.

[gcb70372-bib-0003] Alm, J. , S. Saarnio , H. Nykänen , J. Silvola , and P. Martikainen . 1999. “Winter CO_2_, CH_4_ and N_2_O Fluxes on Some Natural and Drained Boreal Peatlands.” Biogeochemistry 44, no. 2: 163–186. 10.1007/BF00992977.

[gcb70372-bib-0004] Bartoń, K. 2010. “MuMIn: Multi‐Model Inference (Version 1.48.11, p. 1.47.5) [R].” 10.32614/CRAN.package.MuMIn.

[gcb70372-bib-0005] Bates, D. , M. Mächler , B. Bolker , and S. Walker . 2015. “Fitting Linear Mixed‐Effects Models Using lme4.” Journal of Statistical Software 67, no. 1: 1–48. 10.18637/jss.v067.i01.

[gcb70372-bib-0006] Breeuwer, A. , B. J. M. Robroek , J. Limpens , M. M. P. D. Heijmans , M. G. C. Schouten , and F. Berendse . 2009. “Decreased Summer Water Table Depth Affects Peatland Vegetation.” Basic and Applied Ecology 10, no. 4: 330–339. 10.1016/j.baae.2008.05.005.

[gcb70372-bib-0007] Brown, P. J. , and A. T. DeGaetano . 2011. “A Paradox of Cooling Winter Soil Surface Temperatures in a Warming Northeastern United States.” Agricultural and Forest Meteorology 151, no. 7: 947–956. 10.1016/j.agrformet.2011.02.014.

[gcb70372-bib-0008] Bubier, J. L. , A. Costello , T. R. Moore , N. T. Roulet , and K. Savage . 1993. “Microtopography and Methane Flux in Boreal Peatlands, Northern Ontario, Canada.” Canadian Journal of Botany 71, no. 8: 1056–1063. 10.1139/b93-122.

[gcb70372-bib-0009] Bubier, J. L. , P. Crill , A. Mosedale , S. Frolking , and E. Linder . 2003. “Peatland Responses to Varying Interannual Moisture Conditions as Measured by Automatic CO_2_ Chambers.” Global Biogeochemical Cycles 17, no. 2: 2002GB001946. 10.1029/2002GB001946.

[gcb70372-bib-0010] Bubier, J. L. , T. R. Moore , L. Bellisario , N. T. Comer , and P. M. Crill . 1995. “Ecological Controls on Methane Emissions From a Northern Peatland Complex in the Zone of Discontinuous Permafrost, Manitoba, Canada.” Global Biogeochemical Cycles 9, no. 4: 455–470. 10.1029/95GB02379.

[gcb70372-bib-0011] Campbell, J. L. , S. V. Ollinger , G. N. Flerchinger , H. Wicklein , K. Hayhoe , and A. S. Bailey . 2010. “Past and Projected Future Changes in Snowpack and Soil Frost at the Hubbard Brook Experimental Forest, New Hampshire, USA.” Hydrological Processes 24, no. 17: 2465–2480. 10.1002/hyp.7666.

[gcb70372-bib-0012] Cohen, J. L. , J. C. Furtado , M. A. Barlow , V. A. Alexeev , and J. E. Cherry . 2012. “Arctic Warming, Increasing Snow Cover and Widespread Boreal Winter Cooling.” Environmental Research Letters 7, no. 1: 014007. 10.1088/1748-9326/7/1/014007.

[gcb70372-bib-0013] Couwenberg, J. , and H. Joosten . 2005. “Self‐Organization in Raised Bog Patterning: The Origin of Microtope Zonation and Mesotope Diversity.” Journal of Ecology 93, no. 6: 1238–1248. 10.1111/j.1365-2745.2005.01035.x.

[gcb70372-bib-0014] Cresto Aleina, F. , B. R. K. Runkle , T. Brücher , T. Kleinen , and V. Brovkin . 2016. “Upscaling Methane Emission Hotspots in Boreal Peatlands.” Geoscientific Model Development 9, no. 2: 915–926. 10.5194/gmd-9-915-2016.

[gcb70372-bib-0015] Cresto Aleina, F. , B. R. K. Runkle , T. Kleinen , L. Kutzbach , J. Schneider , and V. Brovkin . 2015. “Modeling Micro‐Topographic Controls on Boreal Peatland Hydrology and Methane Fluxes.” Biogeosciences 12, no. 19: 5689–5704. 10.5194/bg-12-5689-2015.

[gcb70372-bib-0016] Dise, N. B. 1993. “Methane Emission From Minnesota Peatlands: Spatial and Seasonal Variability.” Global Biogeochemical Cycles 7, no. 1: 123–142. 10.1029/92GB02299.

[gcb70372-bib-0017] Dise, N. B. , E. Gorham , and E. S. Verry . 1993. “Environmental Factors Controlling Methane Emissions From Peatlands in Northern Minnesota.” Journal of Geophysical Research: Atmospheres 98, no. D6: 10583–10594. 10.1029/93JD00160.

[gcb70372-bib-0018] Dorodnikov, M. , K.‐H. Knorr , Y. Kuzyakov , and M. Wilmking . 2011. “Plant‐Mediated CH_4_ Transport and Contribution of Photosynthates to Methanogenesis at a Boreal Mire: A ^14^C Pulse‐Labeling Study.” Biogeosciences 8, no. 8: 2365–2375. 10.5194/bg-8-2365-2011.

[gcb70372-bib-0019] Dunfield, P. , R. Knowles , R. Dumont , and T. Moore . 1993. “Methane Production and Consumption in Temperate and Subarctic Peat Soils: Response to Temperature and pH.” Soil Biology and Biochemistry 25, no. 3: 321–326. 10.1016/0038-0717(93)90130-4.

[gcb70372-bib-0020] Euskirchen, E. S. , A. D. McGuire , D. W. Kicklighter , et al. 2006. “Importance of Recent Shifts in Soil Thermal Dynamics on Growing Season Length, Productivity, and Carbon Sequestration in Terrestrial High‐Latitude Ecosystems.” Global Change Biology 12, no. 4: 731–750. 10.1111/j.1365-2486.2006.01113.x.

[gcb70372-bib-0021] Fekete, B. M. , D. Wisser , C. Kroeze , et al. 2010. “Millennium Ecosystem Assessment Scenario Drivers (1970–2050): Climate and Hydrological Alterations.” Global Biogeochemical Cycles 24, no. 4: 2009GB003593. 10.1029/2009GB003593.

[gcb70372-bib-0022] Fenner, N. , and C. Freeman . 2011. “Drought‐Induced Carbon Loss in Peatlands.” Nature Geoscience 4, no. 12: 895–900. 10.1038/ngeo1323.

[gcb70372-bib-0024] Frenzel, P. , and E. Karofeld . 2000. “CH_4_ Emission From a Hollow‐Ridge Complex in a Raised Bog: The Role of CH_4_ Production and Oxidation.” Biogeochemistry 51, no. 1: 91–112. 10.1023/A:1006351118347.

[gcb70372-bib-0025] Friborg, T. , T. R. Christensen , and H. Søgaard . 1997. “Rapid Response of Greenhouse Gas Emission to Early Spring Thaw in a Subarctic Mire as Shown by Micrometeorological Techniques.” Geophysical Research Letters 24, no. 23: 3061–3064. 10.1029/97GL03024.

[gcb70372-bib-0026] Ge, M. , A. Korrensalo , R. Laiho , et al. 2023. “Plant Phenology and Species‐Specific Traits Control Plant CH_4_ Emissions in a Northern Boreal Fen.” New Phytologist 238, no. 3: 1019–1032. 10.1111/nph.18798. 36751911

[gcb70372-bib-0027] Ge, M. , A. Korrensalo , A. Putkinen , et al. 2025. “CH_4_ Transport in Wetland Plants Under Controlled Environmental Conditions – Separating the Impacts of Phenology From Environmental Variables.” Plant and Soil 507, no. 1–2: 671–691. 10.1007/s11104-024-06756-x.

[gcb70372-bib-0028] Halmeenmäki, E. , J. Heinonsalo , A. Putkinen , M. Santalahti , H. Fritze , and M. Pihlatie . 2017. “Above‐ and Belowground Fluxes of Methane From Boreal Dwarf Shrubs and *Pinus sylvestris* Seedlings.” Plant and Soil 420, no. 1–2: 361–373. 10.1007/s11104-017-3406-7.

[gcb70372-bib-0029] Heikkinen, J. E. P. , M. Maljanen , M. Aurela , K. J. Hargreaves , and P. J. Martikainen . 2002. “Carbon Dioxide and Methane Dynamics in a Sub‐Arctic Peatland in Northern Finland.” Polar Research 21, no. 1: 49–62. 10.1111/j.1751-8369.2002.tb00066.x.

[gcb70372-bib-0030] Helbig, M. , W. L. Quinton , and O. Sonnentag . 2017. “Warmer Spring Conditions Increase Annual Methane Emissions From a Boreal Peat Landscape With Sporadic Permafrost.” Environmental Research Letters 12, no. 11: 115009. 10.1088/1748-9326/aa8c85.

[gcb70372-bib-0031] Holmgren, M. , C. Lin , J. E. Murillo , et al. 2015. “Positive Shrub–Tree Interactions Facilitate Woody Encroachment in Boreal Peatlands.” Journal of Ecology 103, no. 1: 58–66. 10.1111/1365-2745.12331.

[gcb70372-bib-0032] Hopple, A. M. , R. M. Wilson , M. Kolton , et al. 2020. “Massive Peatland Carbon Banks Vulnerable to Rising Temperatures.” Nature Communications 11, no. 1: 2373. 10.1038/s41467-020-16311-8. PMC7217827 32398638

[gcb70372-bib-0033] IPCC . 2023. Climate Change 2021 – The Physical Science Basis: Working Group I Contribution to the Sixth Assessment Report of the Intergovernmental Panel on Climate Change. 1st ed. Cambridge University Press. 10.1017/9781009157896.

[gcb70372-bib-0034] Ito, A. , T. Li , Z. Qin , et al. 2023. “Cold‐Season Methane Fluxes Simulated by GCP‐CH_4_ Models.” Geophysical Research Letters 50, no. 14: e2023GL103037. 10.1029/2023GL103037.

[gcb70372-bib-0035] Jackowicz‐Korczyński, M. , T. R. Christensen , K. Bäckstrand , et al. 2010. “Annual Cycle of Methane Emission From a Subarctic Peatland.” Journal of Geophysical Research: Biogeosciences 115, no. G2: 2008JG000913. 10.1029/2008JG000913.

[gcb70372-bib-0036] Jentzsch, K. , E. Männistö , M. E. Marushchak , et al. 2024a. “Shoulder Season Controls on Methane Emissions From a Boreal Peatland.” Biogeosciences 21, no. 16: 3761–3788. 10.5194/bg-21-3761-2024.

[gcb70372-bib-0037] Jentzsch, K. , E. Männistö , M. E. Marushchak , et al. 2024b. “Seasonal Chamber Measurements of CH_4_ Fluxes and Pore Water Data From Vegetation Removal Experiments on the Microtopography Scale of Siikaneva Bog, Southern Finland, in 2021 and 2022 [Dataset].” PANGAEA. 10.1594/PANGAEA.971358.

[gcb70372-bib-0038] Joabsson, A. , T. R. Christensen , and B. Wallén . 1999. “Vascular Plant Controls on Methane Emissions From Northern Peatforming Wetlands.” Trends in Ecology & Evolution 14, no. 10: 385–388. 10.1016/S0169-5347(99)01649-3. 10481199

[gcb70372-bib-0040] Karofeld, E. 2004. “Mud‐Bottom Hollows: Exceptional Features in Carbon‐Accumulating Bogs?” Holocene 14, no. 1: 119–124. 10.1191/0959683604hl694rp.

[gcb70372-bib-0041] Karofeld, E. , R. Rivis , H. Tönisson , and K. Vellak . 2015. “Rapid Changes in Plant Assemblages on Mud‐Bottom Hollows in Raised Bog: A Sixteen‐Year Study.” Mires and Peat 16, no. 11: 1–13.

[gcb70372-bib-0042] Kellomäki, S. , M. Maajärvi , H. Strandman , A. Kilpeläinen , and H. Peltola . 2010. “Model Computations on the Climate Change Effects on Snow Cover, Soil Moisture and Soil Frost in the Boreal Conditions Over Finland.” Silva Fennica 44, no. 2: 455. 10.14214/sf.455.

[gcb70372-bib-0043] Kettunen, A. , V. Kaitala , J. Alm , J. Silvola , H. Nykänen , and P. J. Martikainen . 2000. “Predicting Variations in Methane Emissions From Boreal Peatlands Through Regression Models.” Boreal Environment Research 5, no. 2: 115–131.

[gcb70372-bib-0044] Kip, N. , J. F. Van Winden , Y. Pan , et al. 2010. “Global Prevalence of Methane Oxidation by Symbiotic Bacteria in Peat‐Moss Ecosystems.” Nature Geoscience 3, no. 9: 617–621. 10.1038/ngeo939.

[gcb70372-bib-0045] Kokkonen, N. A. K. , A. M. Laine , J. Laine , et al. 2019. “Responses of Peatland Vegetation to 15‐Year Water Level Drawdown as Mediated by Fertility Level.” Journal of Vegetation Science 30, no. 6: 1206–1216. 10.1111/jvs.12794.

[gcb70372-bib-0046] Korpela, I. , R. Haapanen , A. Korrensalo , E.‐S. Tuittila , and T. Vesala . 2020. “Fine‐Resolution Mapping of Microforms of a Boreal Bog Using Aerial Images and Waveform‐Recording LiDAR.” Mires and Peat 26, no. 3: 1–24. 10.19189/MaP.2018.OMB.388.

[gcb70372-bib-0047] Korrensalo, A. , L. Kettunen , R. Laiho , et al. 2018. “Boreal Bog Plant Communities Along a Water Table Gradient Differ in Their Standing Biomass but Not Their Biomass Production.” Journal of Vegetation Science 29, no. 2: 136–146. 10.1111/jvs.12602.

[gcb70372-bib-0048] Korrensalo, A. , I. Mammarella , P. Alekseychik , T. Vesala , and E.‐S. Tuittila . 2022. “Plant Mediated Methane Efflux From a Boreal Peatland Complex.” Plant and Soil 471, no. 1–2: 375–392. 10.1007/s11104-021-05180-9.

[gcb70372-bib-0049] Korrensalo, A. , E. Männistö , P. Alekseychik , et al. 2018. “Small Spatial Variability in Methane Emission Measured From a Wet Patterned Boreal Bog.” Biogeosciences 15, no. 6: 1749–1761. 10.5194/bg-15-1749-2018.

[gcb70372-bib-0050] Kutzbach, L. , D. Wagner , and E.‐M. Pfeiffer . 2004. “Effect of Microrelief and Vegetation on Methane Emission From Wet Polygonal Tundra, Lena Delta, Northern Siberia.” Biogeochemistry 69, no. 3: 341–362. 10.1023/B:BIOG.0000031053.81520.db.

[gcb70372-bib-0039] Laine, J. , H. Vasander , and R. Laiho . 1995. “Long‐Term Effects of Water Level Drawdown on the Vegetation of Drained Pine Mires in Southern Finland.” Journal of Applied Ecology 32, no. 4: 785. 10.2307/2404818.

[gcb70372-bib-0051] Laine, A. , D. Wilson , G. Kiely , and K. A. Byrne . 2007. “Methane Flux Dynamics in an Irish Lowland Blanket Bog.” Plant and Soil 299, no. 1–2: 181–193. 10.1007/s11104-007-9374-6.

[gcb70372-bib-0052] Larmola, T. , E.‐S. Tuittila , M. Tiirola , et al. 2010. “The Role of *Sphagnum* Mosses in the Methane Cycling of a Boreal Mire.” Ecology 91, no. 8: 2356–2365. 10.1890/09-1343.1. 20836457

[gcb70372-bib-0053] Lenth, R. V. 2017. “emmeans: Estimated Marginal Means, Aka Least‐Squares Means (p. 1.10.7) [Computer Software].” 10.32614/CRAN.package.emmeans.

[gcb70372-bib-0054] Li, T. , Y. Huang , W. Zhang , and C. Song . 2010. “CH4MODwetland: A Biogeophysical Model for Simulating Methane Emissions From Natural Wetlands.” Ecological Modelling 221, no. 4: 666–680. 10.1016/j.ecolmodel.2009.05.017.

[gcb70372-bib-0055] Liu, Y. , X. Wang , Y. Wen , H. Cai , X. Song , and Z. Zhang . 2024. “Effects of Freeze‐Thaw Cycles on Soil Greenhouse Gas Emissions: A Systematic Review.” Environmental Research 248: 118386. 10.1016/j.envres.2024.118386. 38316387

[gcb70372-bib-0056] Long, K. D. , L. B. Flanagan , and T. Cai . 2010. “Diurnal and Seasonal Variation in Methane Emissions in a Northern Canadian Peatland Measured by Eddy Covariance.” Global Change Biology 16, no. 9: 2420–2435. 10.1111/j.1365-2486.2009.02083.x.

[gcb70372-bib-0057] Lund, M. , T. R. Christensen , A. Lindroth , and P. Schubert . 2012. “Effects of Drought Conditions on the Carbon Dioxide Dynamics in a Temperate Peatland.” Environmental Research Letters 7, no. 4: 045704. 10.1088/1748-9326/7/4/045704.

[gcb70372-bib-0058] Macdonald, J. A. , D. Fowler , K. J. Hargreaves , U. Skiba , I. D. Leith , and M. B. Murray . 1998. “Methane Emission Rates From a Northern Wetland; Response to Temperature, Water Table and Transport.” Atmospheric Environment 32, no. 19: 3219–3227. 10.1016/S1352-2310(97)00464-0.

[gcb70372-bib-0059] Moore, T. R. , and R. Knowles . 1990. “Methane Emissions From Fen, Bog and Swamp Peatlands in Quebec.” Biogeochemistry 11, no. 1: 45–61. 10.1007/BF00000851.

[gcb70372-bib-0060] Mudryk, L. R. , P. J. Kushner , and C. Derksen . 2014. “Interpreting Observed Northern Hemisphere Snow Trends With Large Ensembles of Climate Simulations.” Climate Dynamics 43, no. 1–2: 345–359. 10.1007/s00382-013-1954-y.

[gcb70372-bib-0061] Nungesser, M. K. 2003. “Modelling Microtopography in Boreal Peatlands: Hummocks and Hollows.” Ecological Modelling 165, no. 2–3: 175–207. 10.1016/S0304-3800(03)00067-X.

[gcb70372-bib-0062] Nykänen, H. , J. Alm , J. Silvola , K. Tolonen , and P. J. Martikainen . 1998. “Methane Fluxes on Boreal Peatlands of Different Fertility and the Effect of Long‐Term Experimental Lowering of the Water Table on Flux Rates.” Global Biogeochemical Cycles 12, no. 1: 53–69. 10.1029/97GB02732.

[gcb70372-bib-0063] Pakarinen, P. 1995. “Classification of Boreal Mires in Finland and Scandinavia: A Review.” Vegetatio 118, no. 1–2: 29–38. 10.1007/BF00045188.

[gcb70372-bib-0086] R Core Team . 2021. R: A Language and Environment for Statistical Computing. R Foundation for Statistical Computing. https://www.R‐project.org/.

[gcb70372-bib-0064] Rantanen, M. , A. Y. Karpechko , A. Lipponen , et al. 2022. “The Arctic has Warmed Nearly Four Times Faster Than the Globe Since 1979.” Communications Earth & Environment 3, no. 1: 168. 10.1038/s43247-022-00498-3.

[gcb70372-bib-0065] Rinne, J. , J.‐P. Tuovinen , L. Klemedtsson , et al. 2020. “Effect of the 2018 European Drought on Methane and Carbon Dioxide Exchange of Northern Mire Ecosystems.” Philosophical Transactions of the Royal Society, B: Biological Sciences 375, no. 1810: 20190517. 10.1098/rstb.2019.0517. PMC7485098 32892729

[gcb70372-bib-0066] Riutta, T. , A. Korrensalo , A. M. Laine , J. Laine , and E.‐S. Tuittila . 2020. “Interacting Effects of Vegetation Components and Water Level on Methane Dynamics in a Boreal Fen.” Biogeosciences 17, no. 3: 727–740. 10.5194/bg-17-727-2020.

[gcb70372-bib-0067] Saarnio, S. , J. Alm , J. Silvola , A. Lohila , H. Nykänen , and P. J. Martikainen . 1997. “Seasonal Variation in CH_4_ Emissions and Production and Oxidation Potentials at Microsites on an Oligotrophic Pine Fen.” Oecologia 110, no. 3: 414–422. 10.1007/s004420050176. 28307231

[gcb70372-bib-0068] Saunois, M. , P. Bousquet , B. Poulter , et al. 2016. “The Global Methane Budget 2000–2012.” Earth System Science Data 8, no. 2: 697–751. 10.5194/essd-8-697-2016.

[gcb70372-bib-0069] Saunois, M. , A. R. Stavert , B. Poulter , et al. 2020. “The Global Methane Budget 2000–2017.” Earth System Science Data 12, no. 3: 1561–1623. 10.5194/essd-12-1561-2020.

[gcb70372-bib-0070] Schimel, J. P. 1995. “Plant Transport and Methane Production as Controls on Methane Flux From Arctic Wet Meadow Tundra.” Biogeochemistry 28, no. 3: 183–200. 10.1007/BF02186458.

[gcb70372-bib-0071] Seppä, H. 2002. “Mires of Finland: Regional and Local Controls of Vegetation, Landforms, and Long‐Term Dynamics.” Fennia ‐ International Journal of Geography 180, no. 1–2: 43–60.

[gcb70372-bib-0072] Strack, M. , J. M. Waddington , M. Turetsky , N. T. Roulet , and K. A. Byrne . 2008. “Northern Peatlands, Greenhouse Gas Exchange and Climate Change.” In Peatlands and Climate Change, edited by M. Strack , 44–69. International Peat Society.

[gcb70372-bib-0073] Ström, L. , and T. R. Christensen . 2007. “Below Ground Carbon Turnover and Greenhouse Gas Exchanges in a Sub‐Arctic Wetland.” Soil Biology and Biochemistry 39, no. 7: 1689–1698. 10.1016/j.soilbio.2007.01.019.

[gcb70372-bib-0074] Ström, L. , A. Ekberg , M. Mastepanov , and T. Røjle Christensen . 2003. “The Effect of Vascular Plants on Carbon Turnover and Methane Emissions From a Tundra Wetland.” Global Change Biology 9, no. 8: 1185–1192. 10.1046/j.1365-2486.2003.00655.x.

[gcb70372-bib-0075] Ström, L. , M. Mastepanov , and T. R. Christensen . 2005. “Species‐Specific Effects of Vascular Plants on Carbon Turnover and Methane Emissions From Wetlands.” Biogeochemistry 75, no. 1: 65–82. 10.1007/s10533-004-6124-1.

[gcb70372-bib-0076] Svensson, B. H. , and T. Rosswall . 1984. “In Situ Methane Production From Acid Peat in Plant Communities With Different Moisture Regimes in a Subarctic Mire.” Oikos 43, no. 3: 341. 10.2307/3544151.

[gcb70372-bib-0077] Tokida, T. , M. Mizoguchi , T. Miyazaki , A. Kagemoto , O. Nagata , and R. Hatano . 2007. “Episodic Release of Methane Bubbles From Peatland During Spring Thaw.” Chemosphere 70, no. 2: 165–171. 10.1016/j.chemosphere.2007.06.042. 17675215

[gcb70372-bib-0078] Treat, C. C. , A. A. Bloom , and M. E. Marushchak . 2018. “Nongrowing Season Methane Emissions–A Significant Component of Annual Emissions Across Northern Ecosystems.” Global Change Biology 24, no. 8: 3331–3343. 10.1111/gcb.14137. 29569301

[gcb70372-bib-0079] Turetsky, M. R. , B. Bond‐Lamberty , E. Euskirchen , et al. 2012. “The Resilience and Functional Role of Moss in Boreal and Arctic Ecosystems.” New Phytologist 196, no. 1: 49–67. 10.1111/j.1469-8137.2012.04254.x. 22924403

[gcb70372-bib-0080] Waddington, J. M. , and N. T. Roulet . 1996. “Atmosphere‐Wetland Carbon Exchanges: Scale Dependency of CO_2_ and CH_4_ Exchange on the Developmental Topography of a Peatland.” Global Biogeochemical Cycles 10, no. 2: 233–245. 10.1029/95GB03871.

[gcb70372-bib-0081] Whittington, P. N. , and J. S. Price . 2006. “The Effects of Water Table Draw‐Down (as a Surrogate for Climate Change) on the Hydrology of a Fen Peatland, Canada.” Hydrological Processes 20, no. 17: 3589–3600. 10.1002/hyp.6376.

[gcb70372-bib-0082] Wilson, D. , J. Alm , T. Riutta , et al. 2007. “A High Resolution Green Area Index for Modelling the Seasonal Dynamics of CO_2_ Exchange in Peatland Vascular Plant Communities.” Plant Ecology 190, no. 1: 37–51. 10.1007/s11258-006-9189-1.

[gcb70372-bib-0083] Yang, Z. , D. Zhu , L. Liu , X. Liu , and H. Chen . 2022. “The Effects of Freeze–Thaw Cycles on Methane Emissions From Peat Soils of a High‐Altitude Peatland.” Frontiers in Earth Science 10: 850220. 10.3389/feart.2022.850220.

[gcb70372-bib-0084] Zhang, T. 2005. “Influence of the Seasonal Snow Cover on the Ground Thermal Regime: An Overview.” Reviews of Geophysics 43, no. 4: 2004RG000157. 10.1029/2004RG000157.

[gcb70372-bib-0085] Zona, D. , B. Gioli , R. Commane , et al. 2016. “Cold Season Emissions Dominate the Arctic Tundra Methane Budget.” Proceedings of the National Academy of Sciences 113, no. 1: 40–45. 10.1073/pnas.1516017113. PMC4711884 26699476

