# Supplementary material for: Seasonality in Diffusive Methane Emissions Differs Between Bog Microforms
Source: Glob Chang Biol. 2025 Jul 25;31(7):e70372. doi: 10.1111/gcb.70372 (PMC12291433; doi:10.1111/gcb.70372)
Supplement: Supplementary file 1 — Data S1. [file GCB-31-e70372-s001.pdf]

Supplementary materials

to

Seasonality in diffusive methane emissions differs between bog microforms

Katharina Jentzsch[1,2], Elisa Männistö[3], Maija E. Marushchak[4,5], Tabea Rettelbach[1,6], Lion Golde[1,7], Aino Korrensalo[5,8], Joshua Hashemi[1], Lona van Delden[1], Eeva-Stiina Tuittila[3], Christian Knoblauch[9,10], Claire C. Treat[1,11]

[1]Alfred Wegener Institute (AWI) Helmholtz Center for Polar and Marine Research, Potsdam, Germany
[2]Institute of Environmental Science and Geography, University of Potsdam, Potsdam, Germany
[3]School of Forest Sciences, University of Eastern Finland, Joensuu, Finland
[4]Department of Biological and Environmental Science, University of Jyväskylä, Jyväskylä, Finland
[5]Department of Environmental and Biological Sciences, University of Eastern Finland, Kuopio, Finland
[6]Institute of Geosciences, University of Potsdam, Potsdam, Germany
[7]Fachbereich III Umweltingenieurwesen – Bau, Berliner Hochschule für Technik, Berlin, Germany
[8]Natural Resources Institute Finland, Joensuu, Finland
[9]Department of Earth System Sciences, University of Hamburg, Hamburg, Germany
[10]Center for Earth System Research and Sustainability, University of Hamburg, Hamburg, Germany
[11]Department of Agroecology, Aarhus University, Aarhus, Denmark

Correspondence to: Katharina Jentzsch (katharina.jentzsch@awi.de)

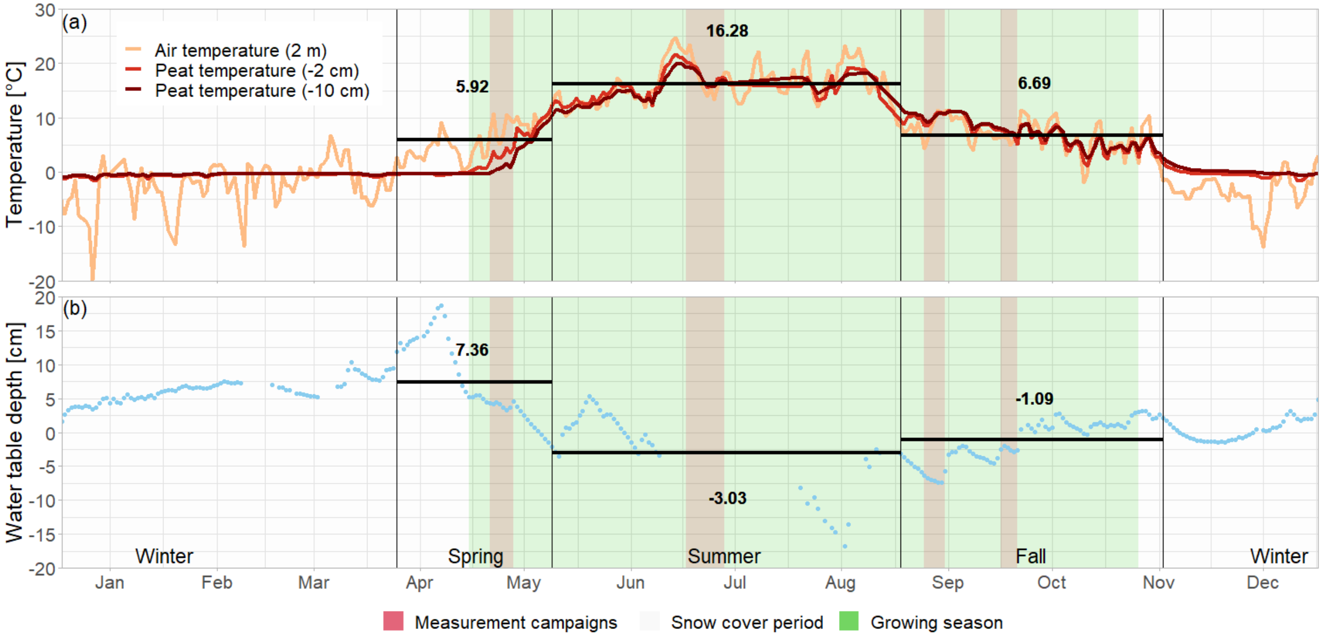

Figure S1: Daily mean air and peat temperatures (a) and WTD (b) at Siikaneva bog in 2022. The snow cover period is the time period between the first and the last day of snow cover even if interrupted by snow-free days. Seasonal mean air temperatures are given as horizontal lines and reported in the figures. We defined the seasons based on thresholds in daily mean temperatures of below 0 °C in winter, between 0 and 10 °C in spring and fall, and above 10 °C in summer (FMI, n.d.). We modified this definition by only recognizing a change between seasons when daily average air temperatures were above the lower threshold (0 °C for spring, 10 °C for summer) or below the upper threshold (10 °C for fall, 0 °C for winter) for at least 3 consecutive days and when periods of consecutive days with average temperatures below the lower or above the upper threshold did not exceed 3 days. The high mean water table in summer is due to large data gaps, especially in July.

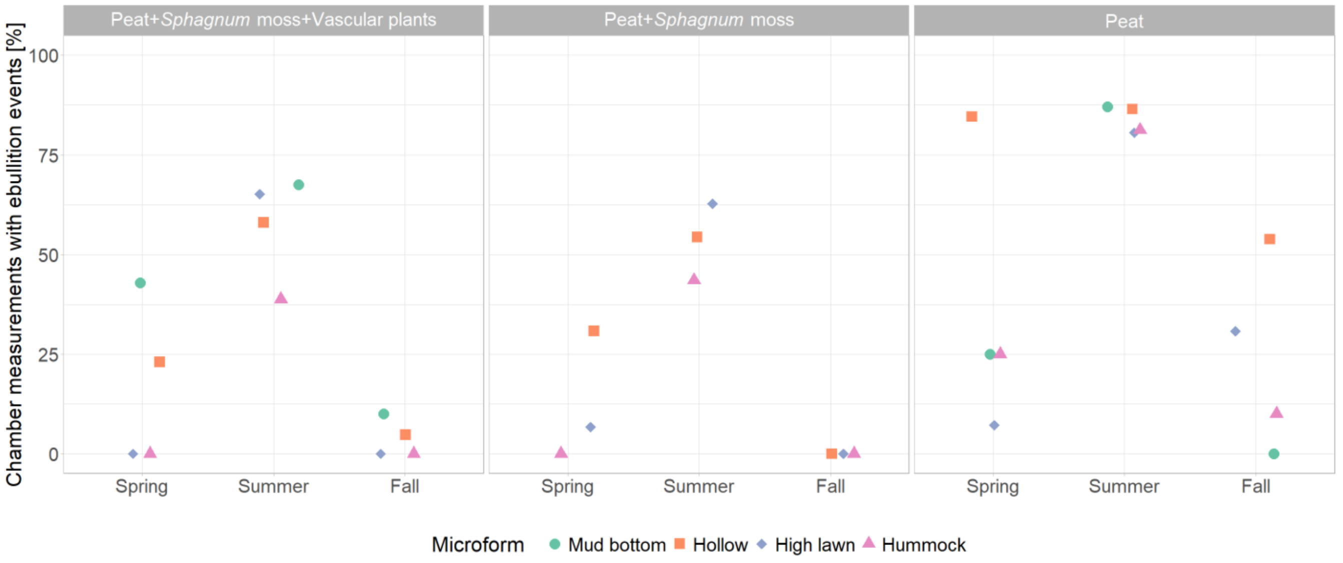

Figure S2: Percentage of measurements with one or more ebullition events by microform, measurement campaign, and vegetation treatment.

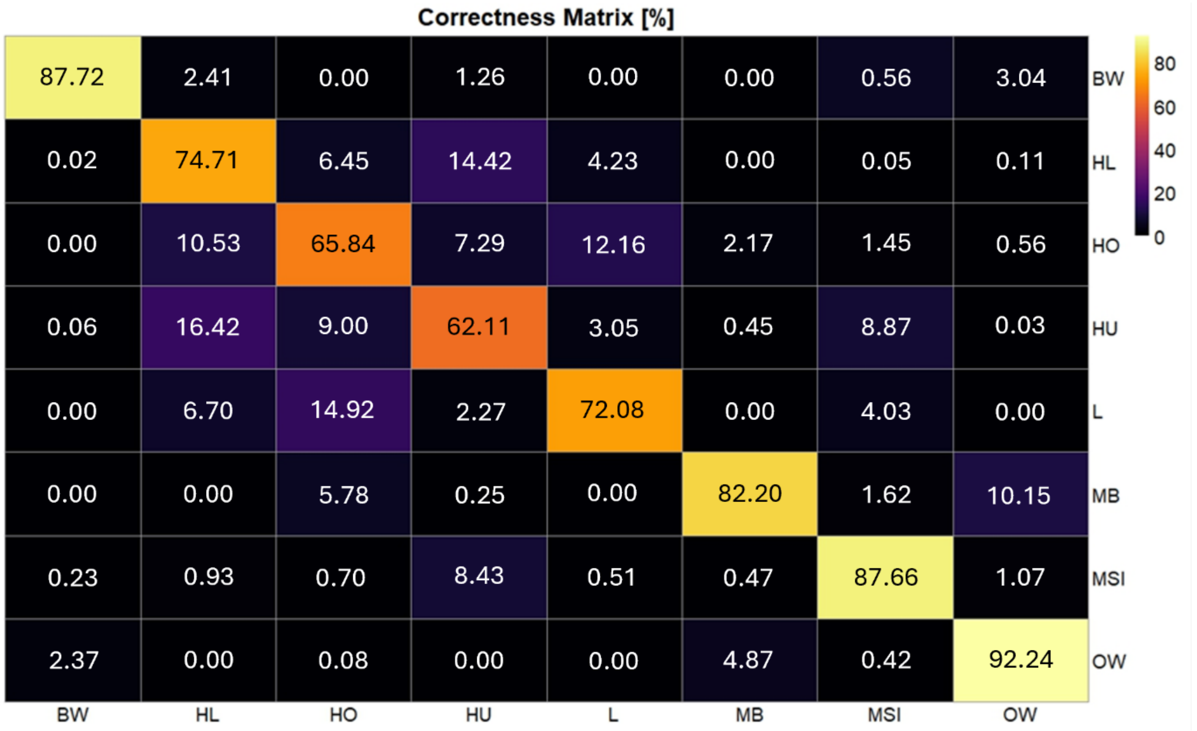

Figure S3: Correlation matrix between predicted and correctly classified land cover classes (board walk (BW), high lawn (HL), hollow (HO), hummock (HU), lawn (L), mud bottom (MB), mineral soil island (MSI), open water (OW)) with the actual class on the x-axis and the predicted class on the y-axis.

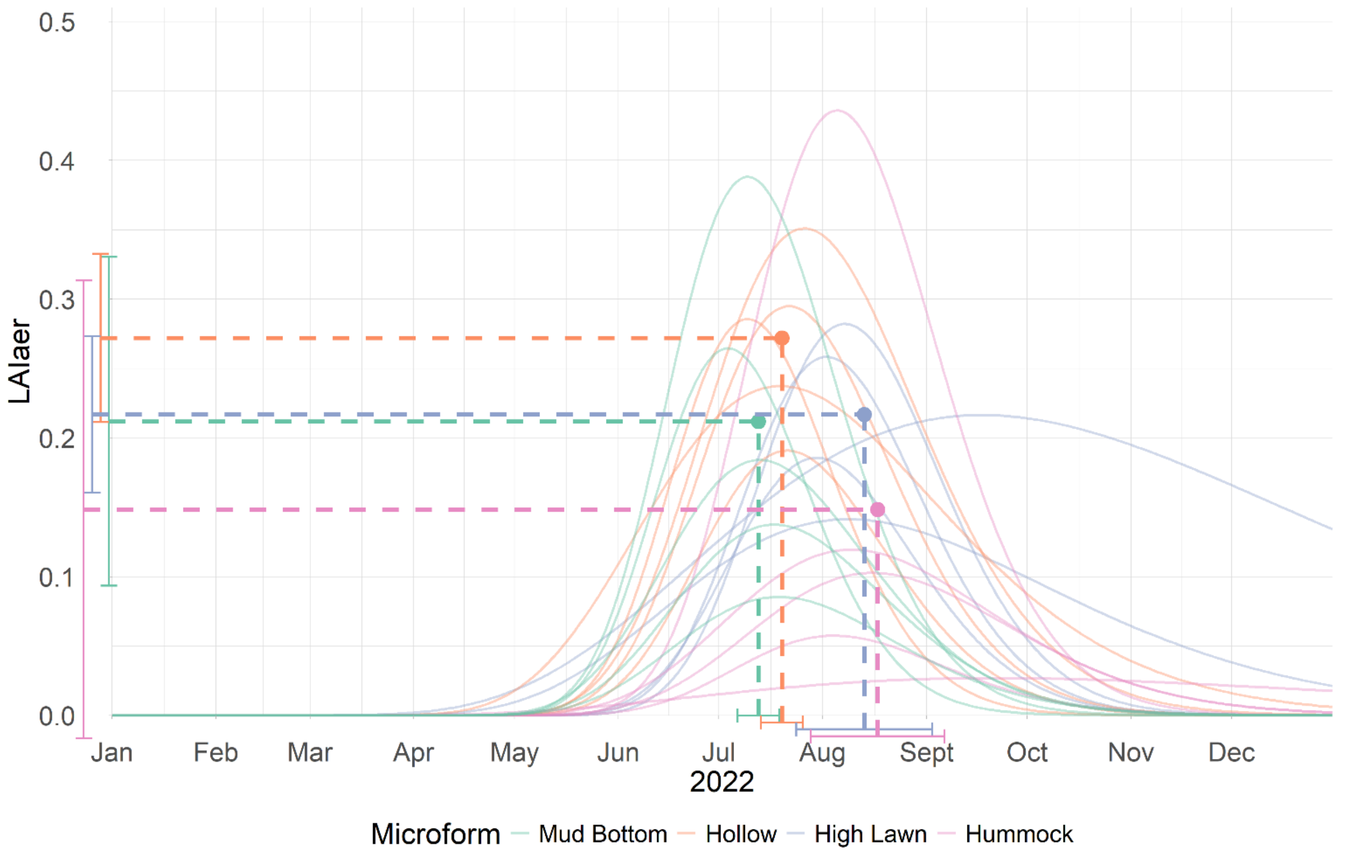

Figure S4: Modelled daily LAIaer by microform. The points indicate the mean day of occurrence of the annual maximum as well as the mean associated maximum LAIaer value. Error bars on the x and y axis give the respective standard deviations.

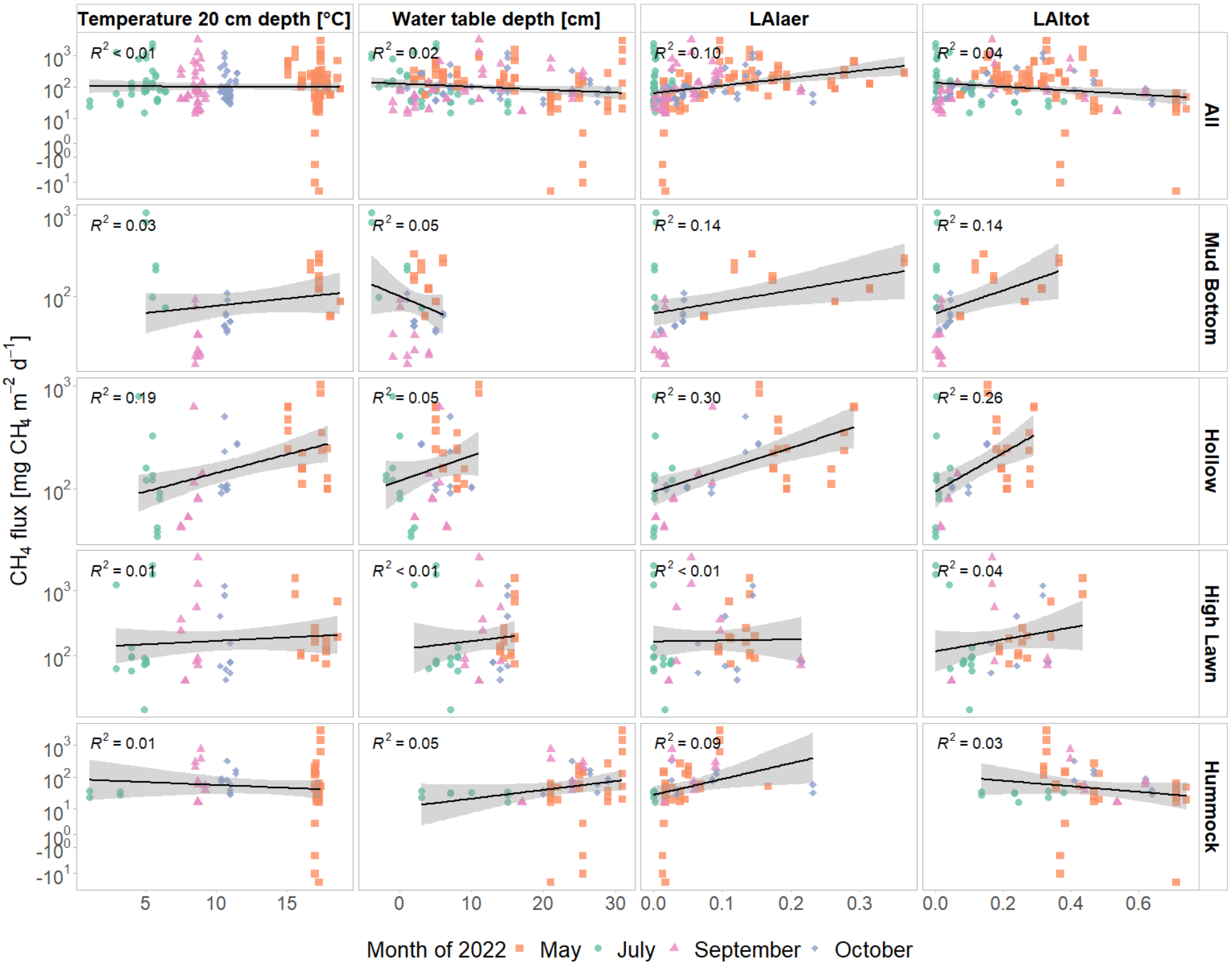

Figure S5: Scatter plots and linear regression of CH_4 fluxes measured at the control plots (PSV) vs. environmental variables, including all data (first row) and separately for each microform (remaining rows). CH_4 fluxes are displayed on a pseudo-logarithmic scale.

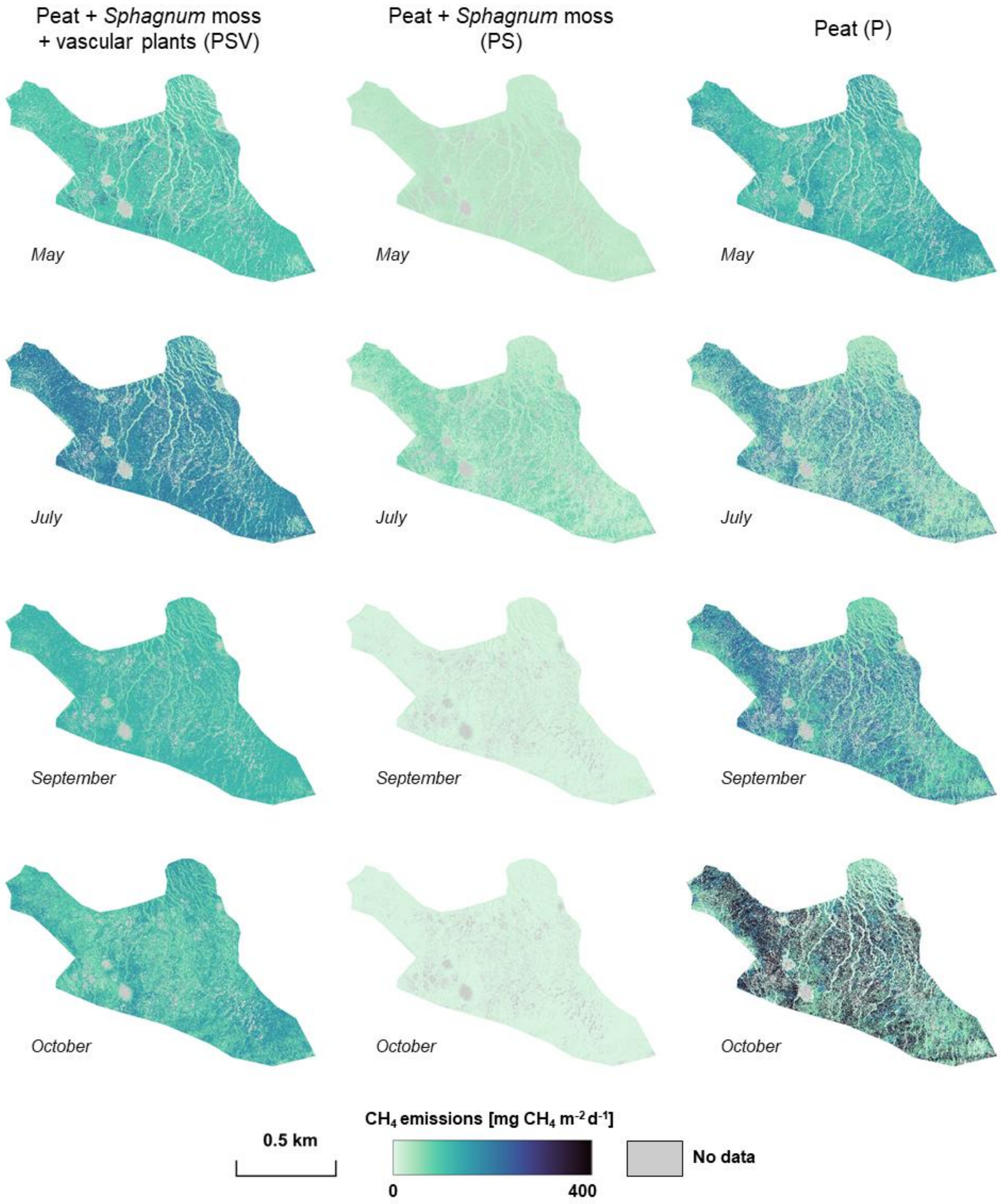

Figure S6: Map of mean CH_4 emissions from Siikaneva bog by season and vegetation treatment based on the microtopography classification in Figure 1.

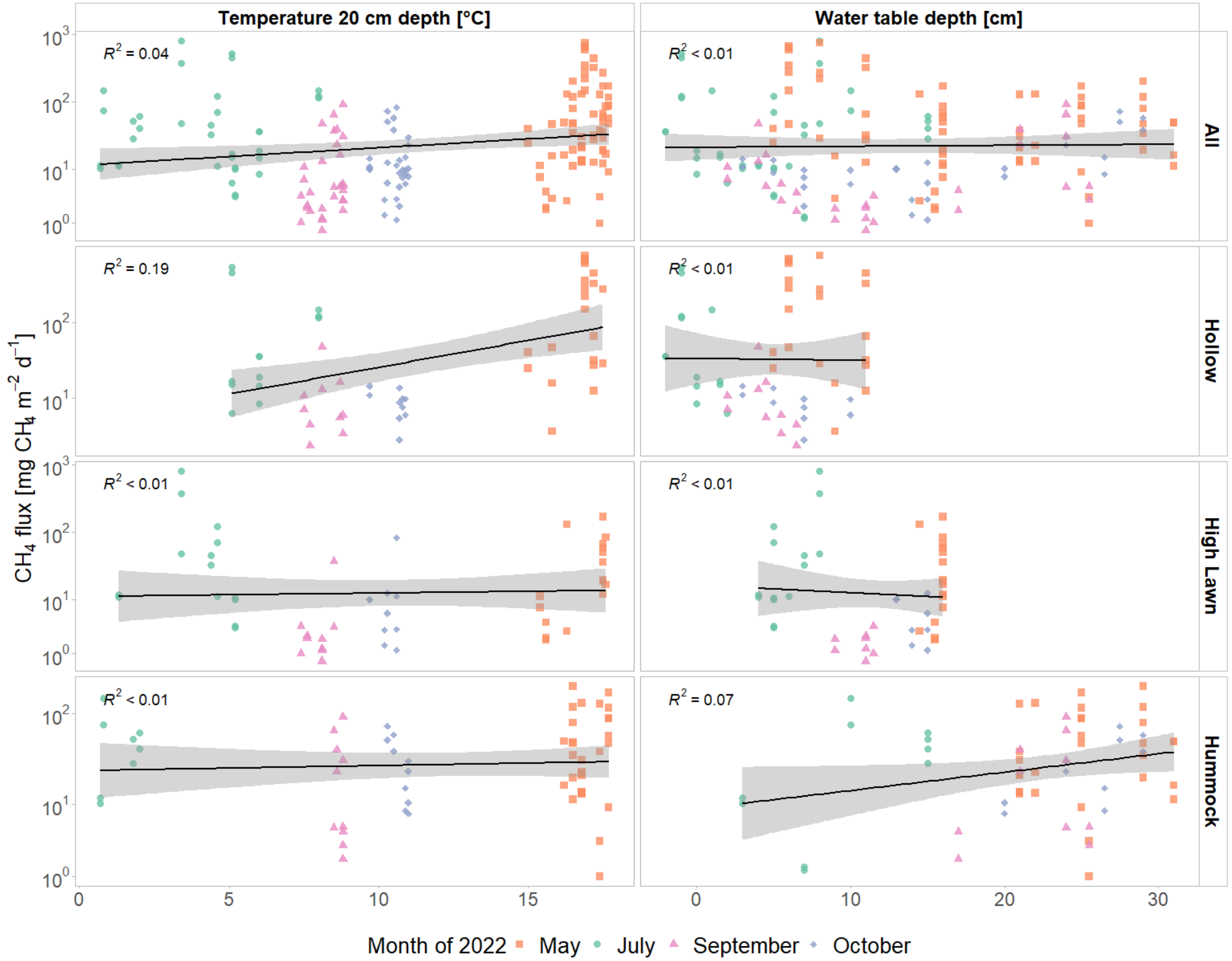

Figure S7: Scatter plots and linear regression of CH_4 fluxes measured at the moss-only plots (PS) vs. environmental variables, including all data (first row) and separately for each microform (remaining rows). CH_4 fluxes are displayed on a pseudo-logarithmic scale.

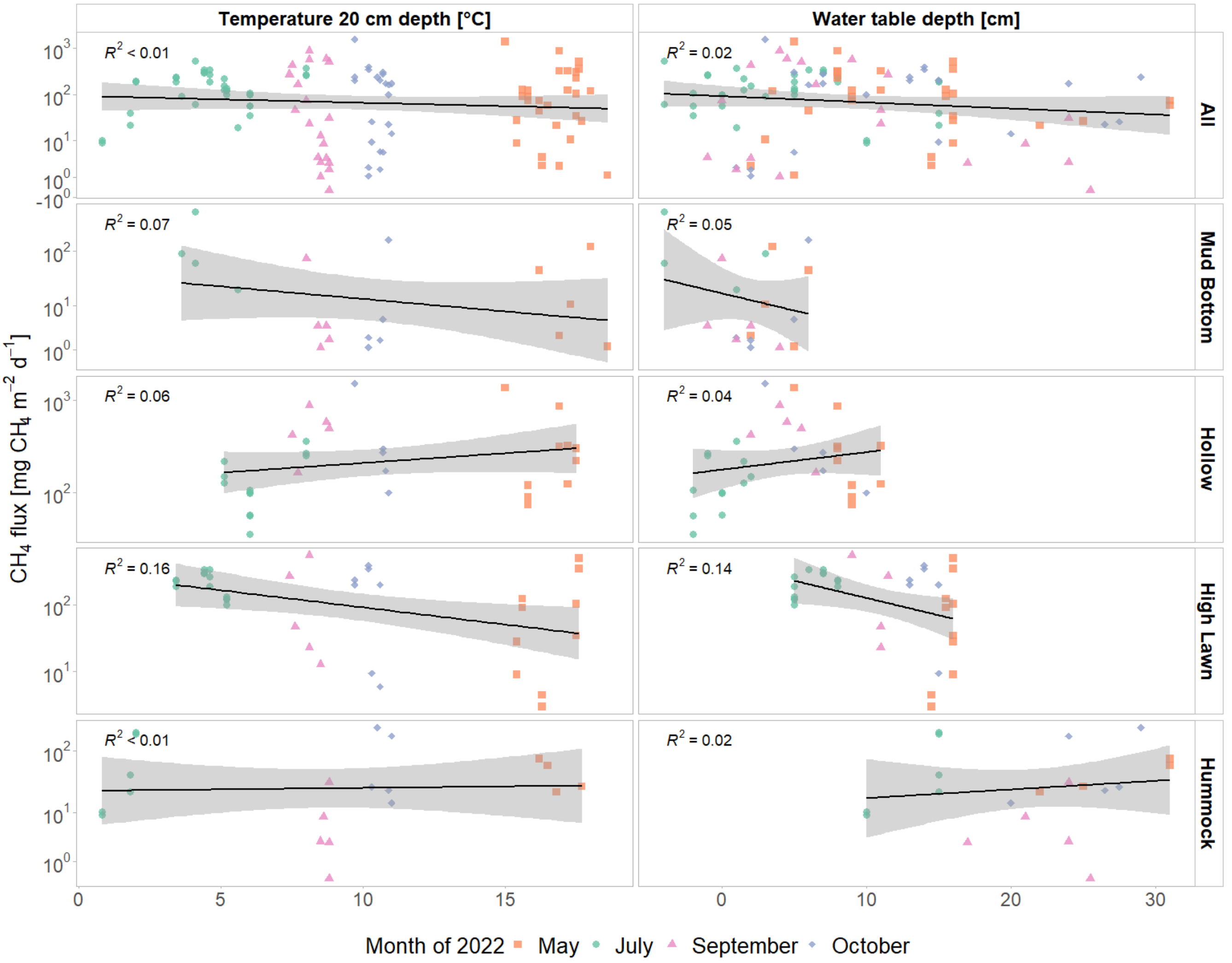

Figure S8: Scatter plots and linear regression of CH_4 fluxes measured at the bare peat plots (P) vs. environmental variables, including all data (first row) and separately for each microform (remaining rows). CH_4 fluxes are displayed on a pseudo-logarithmic scale.

Table S1: Mean cover (%) and standard error (SE) per vascular plant and moss species in the hummock, high lawn, hollow and bare peat surface study plots with intact vegetation at Siikaneva bog measured in 2018.

**Species**	**Vegetation cover (SE)**			
	Mud bottom	Hollow	High lawn	Hummock
Andromeda polifolia		0.1 (0.1)	3.8 (0.7)	0.9 (0.8)
Betula nana			0.2 (0.2)	1.2 (0.2)
Calluna vulgaris			0.02 (0.02)	11.8 (0.02)
Carex lasiocarpa				
Carex limosa	0.2 (0.2)	1.3 (0.4)	0.1 (0.1)	
Carex pauciflora			0.1 (0.1)	0.04 (0.02)
Carex rostrata				
Drosera spp.	0.04 (0.02)	0.1 (0.1)	0.6 (0.1)	0.3 (0.1)
Empetrum nigrum			0.2 (0.2)	2.3 (1.0)
Eriophorum vaginatum			7.8 (2.4)	2.6 (1.4)
*Ledum palustre**				0.1 (0.1)
Menyanthes trifoliata				
*Pinus sylvestris***				0.04 (0.02)
Rhynchospora alba	4.5 (1.5)	1.6 (0.2)	0.1 (0.1)	0 (0)
Rubus chamaemorus			2.3 (1.1)	6.4 (1.6)
Scheuchzeria palustris	0.7 (0.3)	2.6 (0.7)	0.2 (0.2)	0 (0)
Trichophorum cespitosum				
Viola palustris				
Vaccinium oxycoccos	0.02 (0.02)	0.2 (0.1)	2 (1.0)	9.9 (4.8)
**Moss total**	0.2 (0.2)	93.6 (4.7)	99 (0)	97.4 (1.1)
Sphagnum complex '*recurvum*'			6.2 (4.7)	11.6 (4.2)
S. balticum	12.6 (9.0)	12.6 (9.0)	18.2 (18.0)	
S. fuscum			38.0 (14.9)	82.75 (4.8)
*S. magellanicum****			0.2 (0.2)	
S. majus	0.2 (0.2)	82.0 (5.5)		
S. papillosum		15.2 (10.2)	1.0 (1.0)	
S. rubellum			35.6 (15.4)	19.4 (19.4)
Pleurozium schreberi				0.2 (0.2)

* new name: *Rhododendron tomentosum*

** *P. sylvestris* germinant

*** Recently divided into two separate species *S. divinum* Flatberg & K. Hassel and *S. medium* Limpr.

Table S2: Relevant significant differences (*p* <0.05) in CH_4 fluxes between measurement campaigns, microforms (mud bottom (MB), hollow (HO), high lawn (HL), hummock (HU)), and vegetation treatments (control plots (PSV), moss-only plots (PS), bare peat plots (P). Estimate values, standard error (SE), degrees of freedom (DF), and test statistics (*t*-ratio, adjusted *p*-value, and significance level (signif.)) are given as resulting from post-hoc Tukey's HSD test on a linear mixed effects model considering measurement campaign, microtopography, and vegetation treatment as well as their interactions as fixed effects and a unique identified for each measurement plot as random effect. The significance level of the differences is indicated by the number of asterisks as follows: *: $0 < p < 0.001$, **: $0.001 < p < 0.01$, *: $0.01 < p < 0.05$. Flux data was pseudolog-transformed prior to analysis to achieve normality of the residuals.**

Simple contrasts for:	Campaign	Microtopography	Treatment	Value	SE	DF	*t-ratio*	*p*	signif.
**Campaign**	May vs. October	MB	PSV	0.8541	0.244	392	3.504	0.0029	**
	July vs. October	MB	PSV	0.7108	0.204	387	3.492	0.0030	**
	May vs. September	HO	PS	0.7245	0.205	384	3.528	0.0026	**
	May vs. October	HO	PS	0.7416	0.205	384	3.612	0.0019	**
	July vs. September	HO	PS	1.0726	0.190	390	5.634	<0.0001	***
	July vs. October	HO	PS	1.0897	0.190	390	5.724	<0.0001	***
	May vs. September	HL	PS	0.6216	0.203	384	3.069	0.0123	*
	May vs. October	HL	PS	0.9887	0.203	384	4.882	<0.0001	***
	July vs. October	HL	PS	0.7293	0.198	391	3.679	0.0015	**
	May vs. September	MB	P	1.0818	0.338	408	3.205	0.0079	**
	May vs. October	MB	P	1.1315	0.338	408	3.363	0.0047	**
	May vs. July	HL	P	0.6275	0.210	399	2.995	0.0154	*
	May vs. October	HU	P	0.9351	0.305	410	3.068	0.0123	*

	July vs. October	HU	P	0.9888	0.330	391	2.998	0.0153	*
	September vs. October	HU	P	1.0239	0.308	383	3.322	0.0054	**
**Microtopography**	May	MB vs. HU	PSV	0.8984	0.314	173.6	2.865	0.0240	*
	May	HL vs. HU	PSV	0.7975	0.281	124.0	2.839	0.0268	*
	July	HO vs. HU	PSV	0.7578	0.234	62.5	3.243	0.0100	*
	July	HL vs. HU	PSV	0.7217	0.239	68.7	3.014	0.0185	*
	October	MB vs. HL	PSV	-0.8799	0.283	128.6	-3.107	0.0123	*
	July	HO vs. HL	PS	0.7660	0.246	75.1	3.109	0.0138	*
	July	HO vs. HL	P	0.7993	0.284	128.5	2.810	0.0289	*
	September	MB vs. HO	P	-1.6606	0.357	252.3	-4.647	<0.0001	***
	September	MB vs. HL	P	-1.0935	0.339	219.7	-3.228	0.0078	**
	October	MB vs. HO	P	-1.8961	0.357	252.3	-5.306	<0.0001	***
	October	MB vs. HL	P	-1.1091	0.357	252.3	-3.104	0.0114	*
	October	HO vs. HU	P	1.9652	0.357	252.3	5.500	<0.0001	***
	October	HL vs. HU	P	1.1781	0.357	252.3	3.297	0.0061	**
**Treatment**	July	MB	P vs. PSV	-1.0549	0.313	175.7	-3.372	0.0026	**
	September	MB	P vs. PSV	-0.9294	0.322	193.5	-2.883	0.0122	*
	September	HO	P vs. PS	1.5454	0.322	193.5	4.794	<0.0001	***
	September	HO	PS vs. PSV	-1.2797	0.283	128.6	-4.519	<0.0001	***
	October	HO	P vs. PS	1.7448	0.322	193.5	5.412	<0.0001	***
	October	HO	PS vs. PSV	-1.0619	0.283	128.6	-3.750	0.0008	***
	May	HL	P vs. PS	0.7776	0.265	96.9	2.938	0.0114	*
	May	HL	PS vs. PSV	-0.7059	0.257	90.4	-2.745	0.0198	*
	July	HL	P vs. PSV	-0.7276	0.270	108.1	-2.694	0.0221	*
	July	HL	PS vs. PSV	-1.1370	0.254	85.3	-4.470	0.0001	***
	September	HL	P vs. PSV	1.0340	0.302	156.9	3.428	0.0022	**
	September	HL	PS vs. PSV	-1.2923	0.283	128.6	-4.564	<0.0001	***
	October	HL	P vs. PS	1.3634	0.322	193.5	4.229	0.0001	***
	October	HL	PS vs. PSV	-1.8213	0.283	128.6	-6.432	<0.0001	***
	October	HU	P vs. PSV	-1.2888	0.322	193.5	-3.998	0.0003	***
	October	HU	PS vs. PSV	-0.8326	0.283	128.6	-2.940	0.0108	*

Table S3: Relevant significant differences (*p* <0.05) in vegetation effects by plant functional type (PFT) on CH_4 fluxes between measurement campaigns, microforms (mud bottom (MB), hollow (HO), high lawn (HL), hummock (HU), and vegetation treatments (control plots (PSV), moss-only plots (PS), bare peat plots (P). Estimate values, standard error (SE), degrees of freedom (DF), and test statistics (*t*-ratio, adjusted *p*-value, and significance level (signif.)) are given as resulting from post-hoc Tukey's HSD test on a linear mixed effects model considering measurement campaign and microtopography as well as their interactions as fixed effects and a unique identified for each measurement plot as random effect. The significance level of the differences is indicated by the number of asterisks as follows: *: 0 < *p* < 0.001, **: 0.001 < *p* < 0.01, *: 0.01 < *p* < 0.05. Vascular plant effects were pseudolog-transformed prior to analysis to achieve normality of the residuals.**

PFT	Simple contrasts for:	Campaign	Microtopography	Value	SE	DF	*t*-ratio	*p*	signif.
**Vascular plants**	**Campaign**	May vs. October	MB	1.375	0.504	92.2	2.730	0.0374	*
		May vs. September	HU	-1.058	0.398	92.4	-2.661	0.0447	*
		May vs. October	HU	-1.141	0.386	87.0	-2.954	0.0206	*
	**Microtopography**	May	MB vs. HU	1.7955	0.545	76.7	3.295	0.0080	**
		May	HL vs. HU	1.4267	0.427	60.5	3.338	0.0077	**
		July	HO vs. HU	1.1854	0.411	59.6	2.882	0.0274	*
		October	MB vs. HL	-1.4308	0.446	72.0	-3.207	0.0106	*
***Sphagnum* moss**	**Campaign**	May vs. September	HO	-176.706	67.1	72.4	-2.632	0.0497	*
		May vs. October	HO	-208.635	67.1	72.4	-3.108	0.0140	*

Table S4: Upscaling of CH_4 emissions from the measurement plots with intact vegetation (PSV) to the area of Siikaneva covered by the researched microforms by measurement campaign based on mean CH_4 fluxes per microform and campaign using the microtopography map derived from the drone imagery (Table 2). Mean emissions per microform were weighed by the relative areal contribution of the respective microform to the total area of Siikaneva bog. The weighed emissions from all microforms were then summed up to give the total CH_4 emissions from the area of Siikaneva bog that is covered by the researched microform.

Measurement campaign	Microform	Mean CH_4 emission ± standard deviation [mg CH_4 m^{-2} d^{-1}]	Mean CH_4 emission weighed by relative microform contribution to total bog area [mg CH_4 m^{-2} d^{-1}] (% contribution to total emissions)
**May**	Mud bottom	217±243	10±12 (10±13)
	Hollow	112±105	32±31 (32±36)
	Lawn	123±16	16±2 (15±10)
	High lawn	134±189	38±53 (37±57)
	Hummock	29±8	5±2 (5±4)
	Total	115±64*	102±62
**July**	Mud bottom	143±84	7±4 (4±3)
	Hollow	262±194	76±56 (43±38)
	Lawn	231±44	30±6 (17±8)
	High lawn	200±178	56±50 (32±32)
	Hummock	34±77	6±15 (4±9)
	Total	163±64*	175±77
**September**	Mud bottom	54±20	3±1 (2±1)
	Hollow	158±95	46±28 (37±29)
	Lawn	150±10	19±2 (16±7)
	High lawn	143±171	40±48 (33±42)
	Hummock	78±59	15±11 (12±11)
	Total	109±41*	122±57
**October**	Mud bottom	27±17	1±1 (1±1)
	Hollow	92±75	27±22 (22±24)
	Lawn	150±82	19±10 (16±15)
	High lawn	207±307	58±86 (47±79)
	Hummock	93±122	18±23 (14±22)
	Total	107±63*	123±93

* Mean of all microform means weighed by the contribution of the researched microforms to the total area of Siikaneva bog (0.936). These values indicate upscaled emissions assuming equal areal contributions of all microforms to Siikaneva bog or no significant differences in their CH_4 emissions.

References

FMI, n.d. Seasons in Finland - Finnish Meteorological Institute [WWW Document]. URL https://en.ilmatieteenlaitos.fi/seasons-in-finland (accessed 8.24.24).
